# The Role of Environmental Factors on Health Conditions, General Health and Quality of Life in Persons with Spinal Cord Injuries in South Africa

**DOI:** 10.3390/ijerph20095709

**Published:** 2023-05-03

**Authors:** Lucian Bezuidenhout, Anthea Rhoda, David Moulaee Conradsson, Joyce Mothabeng, Conran Joseph

**Affiliations:** 1 Faculty of Community and Health Sciences, University of Western Cape, Cape Town 7701, South Africa; 2 Department of Neurobiology, Care Sciences and Society, Division of Physiotherapy, Karolinska Institutet, 17177 Stockholm, Sweden; 3 Department of Health and Rehabilitation Sciences, Division of Physiotherapy, Stellenbosch University, Cape Town 7602, South Africa; 4 Medical Unit Occupational Therapy & Physiotherapy, Theme Women’s Health and Allied Health Professional, Karolinska University Hospital, 17176 Stockholm, Sweden; 5 Department of Physiotherapy, University of Pretoria, Pretoria 0028, South Africa

**Keywords:** environmental factors, general health, health conditions, quality of life, spinal cord injury, South Africa

## Abstract

Objective: The objective was to describe the individual items of the environmental factors and to investigate the relationship between the environmental factors to health conditions, general health and quality of life in people with SCI in South Africa. Methods: Two hundred persons with SCI participated in a cross-sectional survey design. This study formed part of the International Spinal Cord Injury (InSCI) Community Survey. Four major domains, environmental factors, health conditions, general health and quality of life of the survey questionnaire responses, were used for the analysis. Regression models were used to determine the association between the independent variable, which consisted of the specific environmental factors items, and the dependent variables comprising health conditions, general health and quality of life. Results: The commonly reported environmental barriers were public access, lack of short- and long-distance transport and finances. Environmental factors such as public access (*p* < 0.001), short- (*p* < 0.001) and long-distance transport (*p* = 0.001), and friends’ (*p* = 0.003) and colleagues’ (*p* < 0.001) attitudes and communication (*p* = 0.042) were significantly associated with the presence of secondary health conditions. Finances (*p* = 0.026), family attitudes (*p* = 0.037) and communication (*p* = 0.039) had a significant association with worsened mental health. Services (*p* = 0.022) and communication (*p* = 0.042) were also significantly associated with decreased general health. Conclusion: The results provide insight into modifiable environmental factors policymakers need to consider or adapt to improve the lives of people with SCI in South Africa with respect to health (secondary health conditions), as well as general and mental health.

## 1. Introduction

Spinal cord injury (SCI) is one of the most serious and disabling types of traumatic or non-traumatic injury, which usually affects all aspects of the individual’s life including physiological, psychological and social functions [1,2]. Individuals with SCI face a number of major health conditions, secondary health conditions (e.g., sleep problems, bowel dysfunction and pressure sores), mental health conditions (i.e., depression and happiness) and pain issues, which often have a negative impact on life quality, functioning and emotional well-being, which often leads to preventable premature death [3,4]. People living with SCI also experience activity limitations and participation restrictions in everyday life due to the increased physical demands of living with the SCI [3,5]. For example, results from a British cohort that included 85 persons with SCI, who were injured for more than 20 years, showed that participants had lower physical independence, occupation and social integration, which resulted in decreased participation [6]. Increasing participation for individuals with SCI is important since it can lead to improving mental and physical well-being [7]. The experience of living with a disability is often shaped by environmental factors rather than the presence of living with the disability [8]. This is especially true in low-resource settings such as South Africa, where healthcare is restricted due to a lack of resources, raising various barriers (social and environmental) resulting in challenged reintegration into society for individuals with SCI [9].

In South Africa, data on SCI are rare due to a lack of a national registry and coordinated care systems [10]. However, a study by Joseph et al. [10] provided the first population estimates of the incidence of traumatic spinal cord injury (TSCI), reporting a staggering rate of 75.6 per million person, a figure that is almost the highest globally [10]. A study conducted by Madasa et al. [11] in South Africa revealed that the mortality rate associated with SCI was 24% over a period of four years [11], which highlights the significance of SCI as a public health issue in South Africa. The average age of SCI in South Africa is also documented to be lower compared with developed countries [12], which poses a unique challenge to society and health systems. The younger age of SCI often corresponds to the time of early career development and establishment, and combined with environmental barriers (i.e., transport and inaccessible workplaces) limiting participation, it could further complicate the reintegration of people with SCI into the general society. The South African healthcare system is divided into private and public systems, and access to healthcare services is largely determined by patients’ financial standings. Moreover, the public healthcare system in South Africa has often been documented to have insufficient financial resources, resulting in sub-optimally coordinated and resourced rehabilitation services for individuals with disability [13]. The inadequate rehabilitation services are further elevated by the continuous longstanding nature of SCI rehabilitation considering initial institutional care and discharge back home and into society. However, in situations where specialized rehabilitation units for persons with SCI are available in the public sector, patients have reported that the rehabilitation staff at these centers possess the required expertise [14].

Individuals with SCI may experience physical conditions that can be a direct or indirect result of SCI, such as secondary health conditions (e.g., pain and pressure sores), loss of muscle power and functional limitation (e.g., wheelchair transferring and toileting), which could negatively impact participation in activities (e.g., work, family and social) [15]. The effects of these conditions can extend beyond the person with the SCI and affect the family members as well. Family members may have to take on additional financial and caregiving responsibilities that could lead to relationship and lifestyle changes. These changes can have a negative impact on the health and well-being of both the person with SCI and their family members.

The International Classification of Functioning, Disability and Health (ICF) has been introduced as a conceptualized framework for the role personal and environmental factors have in the construction of the disability [16]. The ICF classifies environmental factors comprising physical, social and attitudinal domains in recognition of the environment’s important role in the everyday functioning of the individual with disabilities [17]. In recent years, an increasing number of studies have focused on understanding participation (i.e., community, physical and social) and factors influencing participation in people with disabilities (e.g., SCI) [3,8,18,19,20].

Environmental factors that vary with physical, social, attitudinal and cultural issues have been documented to be one of the more emphatic factors influencing participation [1], especially in low-resource settings (i.e., South Africa) [21]. Environmental factors negatively impacting people with disabilities often appear as multiple factors simultaneously rather than isolated factors that ultimately have a compounded effect on participation [22]. For example, Cawood et al. [23] determined the environmental barriers and facilitators to participation in 53 people with disabilities (e.g., stroke) in South Africa. They found multiple factors such as the use of technology in daily living, communication, access to transportation, self-perceived mobility and access to buildings to be environmental barriers to participation [23]. In a comparable context, Maart et al. [24] reported similar findings with the addition of educational, climate and labor services as environmental barriers to participation [24]. In a study that relates environmental factors influencing participation in people with SCI in Morocco, Hajjioui et al. [21] showed that individuals with SCI who perceived environmental factors such as lack of public access, financial strain and restricted access to public transportation, public and private places to be barriers also reported more secondary conditions, higher pain intensity, lower mental and general health and quality of life (QoL) [21].

Identifying factors that will overcome physical, social and attitudinal barriers could alleviate the burden on the healthcare system. Moreover, environmental factors negatively affecting people with SCI health conditions, general health and QoL in socio-economically challenged countries such as South Africa are not well described and therefore could lack integrated policy guides and rehabilitation interventions. Therefore, this study aims to describe the individual items of the environmental factors and to investigate the association between environmental barriers with outcomes including health conditions, general health and QoL.

## 2. Materials and Methods

### 2.1. Study Design and Participants

Data were collected using a cross-sectional survey. Two hundred participants, aged ≥18 years and with a confirmed primary diagnosis of either traumatic or non-traumatic SCI, were recruited from private and public healthcare registries in the Western Cape and Gauteng provinces of South Africa. The exclusion criteria were individuals who had a severe cognitive impairment, were hospitalized at the time of data collection, had lower motor neuron paralysis and had inability to give written consent and follow instructions. These exclusion criteria were chosen based on the participant’s understanding of the nature of the study and to increase the generalizability and validity of the study, which could be affected by those hospitalized and with lower neuron paralysis. Participants participated in the International Spinal Cord Injury (InSCI) community survey, of which the primary results of the worldwide data have been published [25,26]. The survey was conducted between January 2017 and May 2019. The InSCI project design, content and protocol are detailed elsewhere [25,26,27]. The South Africa section of the InSCI project was approved by the Biomedical Research Ethics Committee of the University of Western Cape (BMI16/13/24), and all study participants gave written consent prior to participation.

### 2.2. Data Collection

All participants with SCI were invited to one interview session (either in person or by telephone), which consisted of 125 questions from the INSCI community survey. Six participants who conducted the survey over the telephone did the interview over two sessions. The interviews were conducted by two research assistants who were trained in the administration of the questionnaire, specifically, what each item elicits and their response options. The survey instrument was available in the local languages, which were English, Afrikaans and isi-Xhosa (Supplementary File S1), the most widely spoken languages in the provinces that participated. The sociodemographic data included gender, age, disability pension and education level. Years of education before and after SCI were measured in accordance with the International Standard Classification of Education. The SCI characteristics included age and years since injury, etiology, impairment level (paraplegia or tetraplegia) and severity (motor complete or motor incomplete SCI). Four major domains, namely, environmental factors, health conditions, general health and quality of life, of the survey questionnaire responses were used as specific items for the analysis.

#### 2.2.1. Independent Variable (Environmental Factors)

Environmental factors, which consisted of features associated with the support and relationships from others (e.g., family, friends and colleagues), social attitudes (e.g., negative attitude toward people with disability), public services (e.g., availability of short- and long-distance transportation) and policies, were measured as a sum score using the Notwill Environmental Factors Inventory short form (NEFI-SF) [27,28]. The response scores were 1 = not applicable, 2 = no influence, 3 = made my life a little harder and 4 = made my life a lot harder. For simplicity, we reinterpreted the scores by generating the responses of “not applicable” and “no influence” = 0 and 1 = “made my life a little harder” and 2 = “made my life a lot harder” in accordance with Hajjioui et al. [21]. The score range for the environmental factors was 0–28, with a higher score indicating more barriers.

#### 2.2.2. Dependent Variables (Health Conditions, General Health and Quality of Life)

(1) Health conditions related to SCI comprise secondary health conditions, pain intensity and mental health. Secondary health conditions were measured using the modified SCI–Secondary Health Conditions scale [29] and the self-administered comorbidity questionnaire [30]. The response to each item was 1 = no problem to 5 = extreme problem, and we built a sum score over the 14 items ranging from 14 to 70, with a higher score indicating more secondary health problems [21]. Pain intensity was measured using a single item from the Brief Pain Inventory (BPI) with a scale of 0 = no pain to 10 = pain [31]. Mental health (i.e., depression and happiness) was measured using a sub-domain of the short-form health survey (SF-36), and we built a sum score over the five items (5–25), where the response scale for each item was 1 = all of the time to 5 = none of the time in accordance with Ware et al. [32].

(2) General health was assessed using a single item from the SF-36, where participants were asked to rate their health from 1 = poor to 5 = excellent [32].

(3) Quality of life was measured using a single item from the World Health Organization Quality of Life assessment (WHOQoL-BREF), where participants were asked to rate their QoL from 1 = very poor to 5 = very good [33].

### 2.3. Data Analysis

Statistical analyses were carried out using IBM SPSS v.26 software (IBM Corporation, Chicago, IL, USA). Sociodemographic data and SCI characteristics were presented descriptively as numbers (percentages) and mean values (standard deviations). Multiple linear regression models, adjusted for age, the severity of the injury and sex, were used to determine the association between environmental barriers with secondary health conditions, pain intensity and mental health. Multiple logistic regression models, adjusted for age, the severity of the injury and sex, were used to determine the association between environmental barriers with general health and quality of life. *p*-value < 0.05 was seen as significant.

## 3. Results

### 3.1. Participant Characteristics

Table 1 presents the socio-demographic and SCI-related information of the two hundred persons (44 from private and 156 from public registries) with traumatic and non-traumatic SCI. Most SCI participants were male (75%), employed before SCI (61%) and single at the time of the survey (78%), and around half had complete SCI injuries. The average age at SCI onset was 28 (SD: 11) years, with most of the participants also reporting to have experienced an SCI of a traumatic nature, with the prominent causes related to assault and road accidents.

### 3.2. Description of Environmental Factors

**Figure 1:**
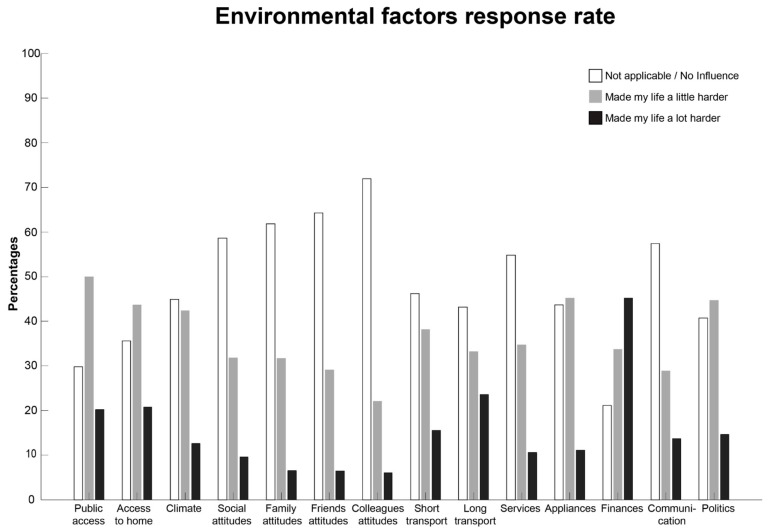
Environmental factors response rate, in percentage.

Figure 1 shows the frequency of the different responses (as percentages) for each of the individual items/questions about the environmental factors. The majority of the individuals responded “not applicable or no influence” on most of the individual environmental factors (Figure 1). The commonly reported barriers perceived to make “life a little bit or a lot harder” were public access, lack of short- and long-distance transport and finances (Figure 1).

### 3.3. Environmental Factors Associated with Health Conditions, General Health and Quality of Life

Table 2 and Table 3 show the relationship between the environmental factors sum score and individual items with health conditions (e.g., secondary health condition, pain intensity and mental health), general health and QoL, which were significantly associated. The environmental factors sum score was significantly associated with secondary health conditions (B = 0.55, *p* < 0.001), pain intensity (B = 0.16, *p* = 0.030), mental health (B = − 0.21, *p* = 0.005) and general health (OR = 0.93, *p* = 0.016). There was no significant association between the environmental sum score and quality of life (Table 3). On an individual item level, environmental factors such as public access (B = 0.30, *p* < 0.001), short (B = 0.33, *p* < 0.001) and long transport (B = 0.25, *p* = 0.001) and friends’ (B = 0.22, *p* = 0.003) and colleagues’ (B=0.27, *p* < 0.001) attitudes and communication (B = 0.15, *p* = 0.042) were significantly associated with the presence of secondary health conditions. Individuals who reported finances (B = −0.22, *p* = 0.026), family attitudes (B = −0.16, *p* = 0.037) and communication (B = −0.16, *p* = 0.039) also had a significant association with worsened mental health (Table 2). Environmental factors such as services (OR = 6.06, *p* = 0.022) and communication (OR = 3.30, *p* = 0.042) were also significantly associated with decreased general health (Table 3). There was no significant association between environmental factors with pain intensity and quality of life. The relationship between all the other environmental factors with health conditions, general health and quality of life are shown in the supplementary material (Tables S1 and S2).

## 4. Discussion

The purpose of this study was to describe the individual items of the environmental factors and to investigate the association between environmental factors with health conditions, general health and quality of life in people with SCI in South Africa. The most commonly reported environmental barriers for people with SCI in South Africa were the lack of short- and long-distance transport and finances. Secondary health conditions had the most significant associations with individual items of the environmental factors, followed by mental and general health.

The impact of a lack of finances on the functioning and quality of life of people living with SCI, specifically in low-resource settings, is evident. Similar to Hajjioui et al. [21], this study found a lack of finances to be associated with a decrease in mental health conditions (feelings of isolation and depression). A low return to work rate could be a major contributing factor to the financial challenges experienced by individuals post-SCI. In this study, 61% of respondents reported being employed prior to the SCI, while only 25% reported being employed post-SCI. Not having a job impacts the financial independence of individuals, which is further exacerbated by the requirements of living with a condition such as an SCI, which often necessitates additional resources and places additional challenges on individuals and their families. It is worth noting that the South African government does provide disability grants (ZAR 1980 per month) to assist individuals with SCI; however, this is often not enough to sustain financial well-being. It has also been reported that the psychological well-being of people with SCI is not directly associated with their injuries [34] but rather formed by daily interaction with their family members and society as a whole [35]. Our results are in line with this, with a decrease in mental health being associated with family attitudes (*p* = 0.026), which individuals reported to make life a lot harder. Providing specifically developed effective communication to individuals with SCI and their family members could play an important role in managing SCI and be a complementary cost-effective rehabilitation service that could promote mental well-being.

Similarly to what was found in this study, the provision of access (public and transport) for people living with disabilities is limited in low-resource settings such as South Africa [8,19]. Individuals with SCI often require specialized transport or adapted vehicles to assist with mobility. This is further amplified by the lack of public rehabilitation centers around South Africa, which results in individuals with SCI often traveling for long distances to attend checkups or appointments for medication using borrowed or hired cars due to the lack of wheelchair-accessible public transport [8]. Although the government in South Africa has tried to improve access to specialized short- and long-distance transport for people with disabilities via a system called “dial a ride”, this is often not ideal as it is not freely available, it needs to be booked well in advance, and reliability of the service is sub-optimal [36]. This lack of availability of appropriate short- and long-distance transport negatively impacts the participation (e.g., societal) and access of these individuals, which could lead to psychological stress and ultimately worsened secondary conditions [21].

South Africa has a large number of social inequalities, with environmental factors (e.g., lack of public transport) and physical public access (i.e., wheelchair ramps) limiting participation for people with disability, especially for those living in rural areas, in the labor market and health-related services, which further enhances the social inequalities [37]. The lack of easily accessible short- and long-distance public transport for people with SCI is also elevated by the lack of knowledge and positive attitudes of public transport operators (i.e., taxi and bus operators) of commuting these individuals in the public sector; therefore, government policies should provide strategies to promote awareness and integration of easy-to-access transport in the public sector for people with disabilities. It is worth noting that such modification of the transport systems and physical environments in low-resource settings such as South Africa is not always possible, and it often depends on legislation changes and financial investments. In addition to these strategies, community-driven approaches founded on social responsibility and accountability should be evoked and supported, rather than a national policy.

The social support network of people with SCI is smaller than the general population [7], with some studies showing that support and positive attitudes toward people with disabilities positively impact their health. Individuals with SCI who do not have a good social support network could adapt their social connections by joining support groups (e.g., online or in person) or participating in SCI-related activities (e.g., adaptive sports or volunteer work), which could result in social interactions and building new relationships [38]. In one of the first studies exploring environmental factors influencing the prevention of secondary health conditions in people with SCI in South Africa, Pilusa et al. [8] found that social support from family, caregivers and peers aided in the prevention of secondary health conditions [8]. Our results are in line with this, with the individuals with SCI who reported that the attitudes of their friends (*p* = 0.003) and colleagues (*p* < 0.001) that “made my life a lot harder” were significantly associated with worsened secondary health conditions. This could be attributed to these individuals moving less to engage socially, which ultimately leads to worsened secondary conditions. Another study by Hosseinigolafshani et al. [39] found that the disability attitude of colleagues, especially in the first years after the SCI event, plays a key role in the formation of their self-belief in their abilities [39]. The general attitudes toward individuals with SCI can be attributed to the lack of public education on the ability of individuals with SCI or general disability in low-resource settings such as South Africa. Therefore, frameworks should be developed to better enhance the public and labor markets’ awareness/perception of people with disabilities (i.e., SCI), and greater accountability measures should be enforced on institutions and systems that exclude people with disabilities.

The impact of environmental factors such as services (i.e., wheelchair training and occupational therapy) and communications (i.e., disability management frameworks) plays an important role in social reintegration and could improve health outcomes in people with SCI. Our results showed that environmental factors such as services (*p* = 0.022) and communications (*p* = 0.042), which individuals with SCI reported “made life a little or a lot harder”, were significantly associated with worsened general health. Providing specialized rehabilitation services (e.g., wheelchair training, promoting functional independence, problem-solving skills and access to assistive devices) in acute and post-acute rehabilitation phases could play an important role in overcoming environmental barriers and improving community reintegration. Although the South African government has developed policies to increase accessibility and equality for individuals with a disability, challenges regarding the accessibility of the environment, transport and lack of recreational facilities continue to persist, with no SCI-specific legislation in place.

Our results also differ from previous studies, which showed that quality of life and pain intensity are associated with environmental factors [19,21]. The difference could be attributed to the sample size, SCI characteristics and socioeconomic status of individuals included in various studies. The study had some limitations, including a relatively small sample size and only having data from two of the nine provinces of South Africa; therefore, the results might not be generalizable to the entire SCI population in South Africa. The data from the INSCI survey were self-reported from a cross-sectional study, which could result in responder bias. The majority of the participants were male (75%); however, this is consistent with other studies on people with SCI [8,19,21]. One of the shortcomings of this study is that certain SCI characteristics such as the level of impairment (i.e., tetraplegic vs. paraplegic), the severity of impairment (complete vs. incomplete) and socioeconomic status (e.g., employment status post-SCI) might influence several outcomes associated with environmental barriers, health conditions, general health and quality of life. Therefore, future work should entail performing multivariate analysis taking into consideration the roles of these different factors on environmental barriers, health conditions, general health and quality of life. Future work should also entail a better distribution (i.e., representative of all nine provinces) of the sample size and include more females with SCI. In addition, more concerted efforts to evaluate and monitor the implementation of policies to support the breakdown of environmental factors to positively impact health conditions, general health and quality of life are needed.

## 5. Conclusions

This is the first study to show modifiable environmental barriers such as public access, transport (short and long distances) and attitudes (family, friends and colleagues) to be significant barriers impacting health conditions and the mental and general health of people with SCI in South Africa. The nature of the environmental factors impacting health transacts various layers of society, which calls for approaches and interventions to be installed from the top (policy) to the bottom (inherent patient factors and family).

## Figures and Tables

**Table 1:** Demographics of participants with spinal cord injury [12].

Characteristics (*n* = 200)	
**Male sex, *n* (%)**	150 (75)
**Age at SCI onset (years), mean (SD) ^a^**	28 (11)
Age ≤ 50 years, *n* (%) ^a^	176 (93)
Age > 50 years, *n* (%) ^a^	13 (7)
**Marital status, *n* (%) ^b^**	
Single	155 (78)
Married/cohabiting	44 (22)
**Employment status before SCI, *n* (%)**	
Working	121 (61)
Not working	79 (39)
**Employment status after SCI, *n* (%)**	
Working	49 (25)
Not working	151 (75)
Level of impairment, *n* (%) *	
Paraplegic	119 (61)
Tetraplegic	77 (39)
Severity of impairment, *n* (%) *	
Complete	102 (52)
Incomplete	94 (48)
**Level of education, *n* (%)**	
Primary	17 (8.5)
Secondary	164 (82)
Short tertiary	10 (5)
Further education	9 (4.5)
Environmental factors, mean (SD) ^b^	9.4 (5.6)
**Health conditions ^b^**	
Secondary conditions	27.7 (8.4)
Pain intensity	3.1 (2.6)
Mental health	15.6 (2.2)
General health, mean (SD)	2.8 (1.0)
Quality of life, mean (SD)	3.5 (0.9)

a = not equal to 200 (*n* = 189), b = not equal to 200 (*n* = 199), * = not equal to 200 (*n* = 196).

**Table 2:** Linear regression model showing the association between environmental factors and health conditions (secondary health conditions, pain intensity and mental health) adjusted for age, sex and severity of SCI.

	Secondary Health Conditions		Pain Intensity		Mental Health	
	B (95% CI)	*p*-Value	B (95% CI)	*p*-Value	B (95% CI)	*p*-Value
**Environmental factors sum score**	0.55 (0.61–0.95)	<0.001	0.16 (0.01–0.13)	0.03	−0.21 (−0.13–−0.02)	0.005
**Environmental factors individual items**						
**Public access**						
No Influence/not applicable	Reference		Reference		Reference	
Made my life a little harder	0.08 (−1.31–4.11)	0.310	−0.02 (−0.76–0.98)	0.808	−0.10 (−1.16–0.33)	0.273
Made my life a lot harder	0.30 (2.94–9.57)	<0.001	0.05 (−0.74–1.40)	0.547	−0.08 (−1.33–0.48)	0.358
**Short transport**						
No Influence/not applicable	Reference		Reference		Reference	
Made my life a little harder	0.08 (−1.18–3.85)	0.297	0.03 (−0.64–1.00)	0.667	−0.07 (−1.03–0.37)	0.353
Made my life a lot harder	0.33 (4.24–11.00)	<0.001	0.08 (−0.54–1.66)	0.317	−0.12 (−1.67–0.20)	0.124
**Long transport**						
No Influence/not applicable	Reference		Reference		Reference	
Made my life a little harder	0.14 (−0.66–4.75)	0.138	−0.02 (−0.99–0.73)	0.771	0.01 (−0.71–0.77)	0.934
Made my life a lot harder	0.25 (1.96–7.89)	<0.001	−0.08 (−1.41–0.47)	0.326	0.04 (−0.59–1.03)	0.593
**Finances**						
No Influence/not applicable	Reference		Reference		Reference	
Made my life a little harder	0.01 (−3.08–3.48)	0.904	−0.13 (−1.69–0.33)	0.187	−0.08 (−1.21–0.51)	0.422
Made my life a lot harder	0.08 (−1.76–4.52)	0.388	−0.12 (−1.58–0.36)	0.217	−0.22 (−1.76–0.11)	0.026
**Family attitudes**						
No Influence/not applicable	Reference		Reference		Reference	
Made my life a little harder	0.02 (−2.27–2.93)	0.805	0.00 (−0.83–0.79)	0.955	−0.03 (−0.81–0.56)	0.714
Made my life a lot harder	0.12 (−0.98–8.80)	0.116	0.04 (−1.11–1.93)	0.598	−0.16 (−2.66–−0.09)	0.037
**Friends’ attitudes**						
No Influence/not applicable	Reference		Reference		Reference	
Made my life a little harder	0.00 (−2.57–2.65)	0.974	0.02 (−0.75–0.91)	0.844	−0.11 (−1.20–0.20)	0.161
Made my life a lot harder	0.22 (2.49–12.07)	0.003	0.03 (−1.26–1.78)	0.738	−0.14 (−2.49–0.08)	0.065
**Colleagues’ attitudes**						
No Influence/not applicable	Reference		Reference		Reference	
Made my life a little harder	0.16 (0.46–6.01)	0.023	0.03 (−0.71–1.08)	0.682	−0.12 (−1.38–0.14)	0.112
Made my life a lot harder	0.27 (4.47–14.18)	<0.001	0.08 (−0.76–2.38)	0.308	−0.10 (−2.28–0.39)	0.162
**Communication**						
No Influence/not applicable	Reference		Reference		Reference	
Made my life a little harder	−0.03 (−3.34–2.14)	0.667	−0.12 (−1.54–0.17)	0.113	−0.16 (−1.49–−0.04)	0.039
Made my life a lot harder	0.15 (0.14–7.28)	0.042	−0.02 (−1.24–1.00)	0.827	−0.02 (−1.07–0.82)	0.791

**Table 3:** Logistic regression model showing the association between environmental factors and general health and quality of life adjusted for age, sex and severity of SCI.

	General Health		Quality of Life	
	OR (95% CI)	*p*-Value	OR (95% CI)	*p*-Value
**Environmental factors sum score**	0.93 (0.88–0.99)	0.016	0.93 (0.87–1.01)	0.066
**Environmental factors individual items**				
**Services**				
No Influence/not applicable	Reference		Reference	
Made my life a little harder	1.23 (0.63–2.40)	0.541	0.99 (0.33–2.95)	0.985
Made my life a lot harder	6.06 (1.29–28.38)	0.022	2.32 (0.63–8.47)	0.204
**Communication**				
No Influence/not applicable	Reference		Reference	
Made my life a little harder	0.97 (0.48–1.96)	0.940	0.59 (0.18–1.90)	0.373
Made my life a lot harder	3.30 (1.05–10.41)	0.042	0.23 (0.03–1.82)	0.163

## Data Availability

Data are available upon request from the corresponding author.

## Supplementary Materials

The following supporting information can be downloaded at: https://www.mdpi.com/article/10.3390/ijerph20095709/s1, File S1: International Spinal Cord Injury Survey (InSCI) in Xhosa and Afrikaans languages; Table S1: Linear Regression model showing the association between Environmental factors with Health conditions (Secondary health conditions, Pain Intensity and Mental Health) adjusted for age, sex and severity of SCI. Table S2: Logistic Regression model showing the association between Environmental factors with General Health and QoL adjusted for age, sex and severity of SCI.
