# Supplementary material for: The Role of Environmental Factors on Health Conditions, General Health and Quality of Life in Persons with Spinal Cord Injuries in South Africa
Source: Int J Environ Res Public Health. 2023 May 3;20(9):5709. doi: 10.3390/ijerph20095709 (PMC10178437; doi:10.3390/ijerph20095709)
Supplement: Supplementary file 1 [file ijerph-20-05709-s001.zip › ijerph-2310147-supplementary-File S1.pdf]

International Spinal Cord Injury Survey (InSCI)

The first worldwide survey
on community-dwelling persons with spinal cord injury.

Countries all over the world take part in this initiative to capture the experience of living with spinal cord injury by asking those who know best: persons with spinal cord injury.

A collaboration of

ISPRM International Society of Physical and Rehabilitation Medicine

World Health Organization

ISCOS The International Spinal Cord Society

Dear participant

Welcome to the InSCI survey, we are very happy to have you on board!

InSCI is the first worldwide survey on community-dwelling persons with spinal cord injury. Countries all over the world take part in this initiative to capture the experience of living with spinal cord injury by asking those who know best: persons with spinal cord injury.

Please fill in the questionnaire as completely as possible and don't leave any questions unanswered. There is no right or wrong and no good or bad answer. It is important that you answer spontaneously and decide which response best applies to your personal situation.

You can also complete the questionnaire online at [www.insci.com]. Please login with your InSCI-ID and your personal password:

Your InSCI-ID is: ######

Your personal password is: ######

We guarantee that your data is protected with the highest security standards. No personal data will be handed out to third persons outside the study center. All questionnaires are anonymized by a unique identification number (InSCI-ID) and there is no personal information such as name or address on the paper or online questionnaire.

In case you have any question or need support in questionnaire completion, we are happy to help. Please send us an email at contact@en.insci.network or contact our toll-free InSCI-helpline at 0700 523 696 631.

Thank you again for your commitment!

Your InSCI-Team [may add names of local PIs]

Iinkcukacha zoMntu

1. Nceda uchaze isini sakho:

- ○ Indoda
- ○ Ibhinqa

2. Ingaba wawuzelwe ngoluphi usuku, inyanga nonyaka?

USUKU/ INYANGA/ UNYAKA

□□/□□/□□□□

3. Ingaba wawuzalelwe kweliphi ilizwe?

✎..

4. Ingaba utshatile?

- ○ Anditshatanga
- ○ Nditshatile
- ○ Siyahlalisana okanye liqabane
- ○ Wahlukene okanye uqhawule umtshato
- ○ Uhlolokazi

5. Ngubani ohlala nawe ekhaya?

Krwela zonke ezingenayo

- □ Ndihlala ndodwa
- □ Nabantwana abangaphantsi kweminyaka eli-14 ubudala, inani labo: ✎...............
- □ Ulutsha oluphakathi kweminyaka eli-14 neli-18 ubudala, inani lalo: ✎................
- □ Abantu abaphakathi kweminyaka eli-18 nama-64 ubudala, inani labo: ✎................
- □ Abantu abangaphezulu kwama-64 ubudala, inani labo: ✎................
- □ Ndihlala kwindawo ekhethekileyo *umz. ikhaya labantu abadala, ikhaya lonyango ngoomongikazi*

6. Ingaba uyalufumana uncedo ngezinto zakho ozenzayo zemihla ngemihla ekhaya okanye ngaphandle?

- ○ Hayi
- ○ Ewe, ngaba bantu balandelayo:

  Krwela zonke ezingenayo

  - □ Usapho
  - □ Abahlobo
  - □ Abaqeqeshiweyo okanye abancedisi abahlawulwayo

7. Lithini ibakala eliphezulu lemfundo oligqibileyo? [*iintlobo ezikhethekileyo ngokwelizwe*]

- ○ Eliphantsi
- ○ Elisezantsi
- ○ Eliphezulu
- ○ Elingaphaya kweSekondari
- ○ Efutshane yamaziko aphezulu
- ○ Isidanga okanye okulinganayo
- ○ Imastazi okanye okulinganayo
- ○ Okunye, kuchaze: ✎ ....

8. Mingaphi iminyaka yemfundo okanye eyoqeqesho othe walugqiba?

Iminyaka yemfundo okanye eyoqeqesho ngaphambi kokuba ufumane ingozi yomnqonqo: ✎......... *(Inani leminyaka)*

Iminyaka yemfundo okanye eyoqeqesho emva kokuba ufumane ingozi yomnqonqo: ✎............ *(Inani leminyaka)*

9. Xa uthabathela ingqalelo bonke abantu ohlala nabo ekhayeni lakho abasebenzela umvuzo okanye intlawulo: ingaba ithini ingeniso iyonke yekhaya [*ngaphambi, emva*] kweerhafu ngenyanga umyinge?

- ○ < R1100 ngenyanga
- ○ R1101 – R3000 ngenyanga
- ○ R3001 –R4500 ngenyanga
- ○ R4501 – R6000 ngenyanga
- ○ R6001 –R9000 ngenyanga
- ○ R9001 – R12000 ngenyanga
- ○ R12001 – R20 000 ngenyanga
- ○ R20001 – R3000 ngenyanga
- ○ R30001 – R50000 ngenyanga
- ○ > R500001

10. Cinga ngale leli njengemele apho abantu bami khona e*[ilizwe].*

Kwincopho yeleli ngabo bantu abazizityebi – abo banemali eninzi, abo bafundileyo kwaye bakwimisebenzi ehlonitshwayo. Ezantsi ngabo bantu bahlupheke kakhulu – abo banemali encinane, imfundo ephantsi, kwaye bakwimisebenzi ejongelwe phantsi okanye abaphangeli. Xa usiya unyuka kwileli, uya kusondela kwabo bantu basencotsheni; xa usiya ezantsi, uya kuba kufutshane naba abasezantsi.

Ingaba ungazibeka ndawoni wena kule leli?

Nceda ufake u- **X** kwinqwanqwa apho wena unokuzibeka kulo ngoku ebomini bakho, xa uzithelekisa nabanye abantu *[kwilizwe lakho]*

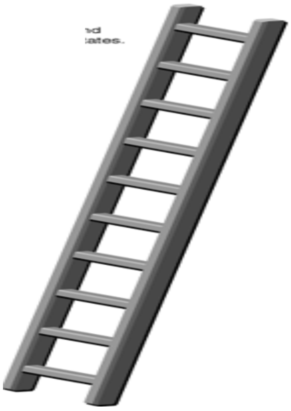

Iimpawu zomonakalo

11. Nceda uchaze inqanaba lomonzakalo kumnqonqo wakho:

- ○ Ukufa amanqe (intshukumo nemvakalelo eqhekelileyo kumalungu angezantsi)
- ○ Ukufa amalungu omzimba onke (ukungabikho okanye imvakalelo eyahlukileyo kwentshukumo okanye imvakalelo kwiingalo okanye imilenze)

12. Ingaba umonzakalo wakho ugqibelel okanye awugqibelelanga?

- ○ Ugqibelele (andikwazi kuva nokushukumisa naliphi na elinye ilingu lomzimba ongezantsi kwale ndawo yomonzakalo)
- ○ Awugqibelelanga (andikwazi ukuva nakushukumisa amanye amalungu omzimba ongezantsi kwale ndawo yomonzakalo)

13. Nceda ucacise ukuba yintoni unobangela womonzakalo wakho kumnqonqo

Okwenziwe yingozi:

Jonga konke okungqameleneyo

Uumzekelo xa ujonga ibhokisi 'umonzakalo ngexesha lomsebenzi', nceda cacisa ukuba ingaba kukuwa okanye omnye unobangela wengozi.

- ☐ Umonzakalo ngexesha lezemidlalo
- ☐ Umonzakalo ngexesha lolonwabo
- ☐ Umonzakalo ngexesha lomsebenzi
- ☐ Ingozi yemoto
- ☐ Umonzakalo ngenxa yobondlobongela (e.g., isilonda sokudutyulwa)
- ☐ Ukuwa ngaphantsi kwemitha enye
- ☐ Ukuwa ngaphezulu kwemitha enye
- ☐ Omnye unobangela womonzakalo: ✎ ....

Unobangela osisifo:

Krwela okubandakanyekayo

- ☐ Ukuyekela komqolo
- ☐ Ithumba – elingenabungozi
- ☐ Ithumba – elinobungozi (umhlaza)
- ☐ Ingxaki yemithambo (umz., e.g., iskemiya, ukopha, ukungemi kakuhle)
- ☐ Ukusuleleka (umz.,ibhkathiriya, iintsholongwane)
- ☐ Ezinye izifo: ✎ ....

14. Nceda uchaze ngokuchanekileyo kangangoko ukuba wawenzakale ngawuphi umhla umnqonqo wakho:

USUKU/ INYANGA/ UNYAKA

☐☐/☐☐/☐☐☐☐

Udlamko nemvakalelo

Lemibuzo imalunga nokuba waziva njani kwaye izinto zabanjani kuwe kwezi veki zine zidlulileyo. Nceda kumbuzo nganye unike impendulo iyeleleneyo nendlela oziva ngayo.

Lixesha elingakanani kwezi veki zine zidlulileyo ...		*Ngalo lonke ixesha*	*Amaxesha amaninzi*	*Ngelinye ixesha*	*Ixesha elincinci*	*Akukho xesha ndiziva njalo*
15.	Ingaba uziva udlamkile?	○	○	○	○	○
16.	Wakhe waxhalaba kakhulu?	○	○	○	○	○
17.	Wakhe waziva udakumbile, ubone ukuba akukho nto inokwenza udlamke?	○	○	○	○	○
18.	Ukhe waziva upholile kwaye useluxilweni? ?	○	○	○	○	○
19.	Ubukhe udlamke kakhulu?	○	○	○	○	○
20.	Wakhe waziva udakumbile kwaye ubuthakathaka?	○	○	○	○	○
21.	Uziva uphelelwa ngamandla?	○	○	○	○	○
22.	Wakhe wonwaba?	○	○	○	○	○
23.	Uziva udiniwe?	○	○	○	○	○

Ingxaki zempilo

Ngezi ngxaki zilandelayo zempilo, nceda uthelekelele ukuba ezi ngxaki zibe ziingxaki ezinjani kwezi nyanga zintathu ziggithileyo. Ukuba uthe wazifumana ezi ngxaki zempilo, nceda uphawule ukuba ingaba uthe wafumana unyanga okanye hayi (umzekelo ukusela amayeza okanye ukufumana unyango loogqirha okanye abanye abaqeqeshelwe ezempilo).

	1 Akukho Ngxaki	2	3	4	5 Ingxaki enkulu	Ukhe/ wakhe wafumana unyango lwayo?
**24. Ingxaki zokulala**<br>*umzekelo, ingxaki zokwehla kobuthongo okanye ulala ubusuku bonke uvuke ekuseni kakhulu.*	○	○	○	○	○	○ Ewe<br>○ Hayi
**25. Ukuhambi kakuhle kwesisu**<br>*umzekelo, urhudo,ungakwazi ukubamba ilindle ('ingozi') nokuqhina.*	○	○	○	○	○	○ Ewe<br>○ Hayi
**26. Usuleleko lomchamo**<br>*Umzekelo, izintso okanye ukusuleleka kwesinyi*	○	○	○	○	○	○ Ewe<br>○ Hayi
**27. Isinyi esingasebenzi kakuhle**<br>*umzekelo,ukuzichamela ('iingozi'), isinyi okanye isinyi okanye amaqhuma kwizintso, ingxaki kwizintsho, umchamo ongavakali xa uphuma and umchamo ugcinakele.*	○	○	○	○	○	○ Ewe<br>○ Hayi
**28. Ukuphela kwemizwa kwezesondo**<br>*umzekelo.,ukuphelelwa yimizwa yesondo, ukuvukelwa,ubumanzi, nokufikelela kukwaneliseka ngokwesondo.*	○	○	○	○	○	○ Ewe<br>○ Hayi
**29. Isimo sokwehlisa okanye ukuqinisa izihlunu**<br>*Oku kukungakwazi ukusebenzisi amalungu omzimba ngokupheleleyo kwimidibaniso yamalungu omzimba.*	○	○	○	○	○	○ Ewe<br>○ Hayi
**30. Inkantsi yezihlunu, ukuqinelwa zizhlunu**<br>*Oku kubhekisa kwiintshukumo zezihlunu ezingalawulekileyo ezinjengokushuma kwezihlunu ngokungalawulekiyo okanye inkantsi yezihlunu.*	○	○	○	○	○	○ Ewe<br>○ Hayi
**31. Izilonda ngenxa yokuhlala ndawonye, amatyhungutyhungu**<br>*Ezi zilonda zivela njengerhashalala yesikhumba okanye ububomvu kwaye isenokuqhubekeka ibesisilonda esinobumdaka esingapholiyo.*	○	○	○	○	○	○ Ewe<br>○ Hayi
**32. Iingxako zokuphefumla**<br>*Iimpawu neengxaki zokwasuleleka ziquka iingxaki zokuphefufumla nokunyuka kwemikhunya.*	○	○	○	○	○	○ Ewe<br>○ Hayi
**33. Umonzakalo obangelwe kukulahleka**	○	○	○	○	○	○ Ewe<br>○ Hayi

	**kwemvakalelo**<br>*Umzekelo, izilonda zokutsha ezinololwelo olushushu okanye ukuhlala phantsi ixesha elide kufutshane nehitha okanye umlilo.*						
**34.**	**Iingxaki zokuhamba kwegazi**<br>*Oku kuquka ukudumba kwemithambo, iinyawo, imilenze okanye izandla, okanye uukwenzeka kwamahlwili egazini.*	○ *1 Akukho ngxaki*	○ *2*	○ *3*	○ *4*	○ *5 Ingxaki inkulu kakhulu*	○ Ewe<br>○ Hayi<br>*Ukhe/ wakhe wafumana unyango lwayo*
**35.**	**Ukunyukelwa luxinizelelo lwegazi ngokukhawuleza**<br>*Iimpawu zokhawuleza kunyuke uxinizelelo lwegazi nokubila, amabala kwisikhumba, iingongoma, ukungabini owexeshana nentloko ebuhlungu.*	○	○	○	○	○	○ Ewe<br>○ Hayi
**36.**	**Ukufutheka ngenxa yokuma ixesha elide**<br>*Oku kuquka imvakalelo yokuba nesiyezi kulandela ukutshintsha isikhundla sokuma. Oku kubangelwa kukuhla koxinizelo lwegazi.*	○	○	○	○	○	○ Ewe<br>○ Hayi
**37.**	**Intlungu**<br>*Ukuba neentlungu kubomi bemihla ngemihla.*	○	○	○	○	○	○ Ewe<br>○ Hayi

38. Nceda uthelekele intlungu yakho ngokuthi urhangqe inombolo echaza ngcono intlungu yakho xa ibiphezulu kule veki iphekileyo.

Akukho ntlungu ←→ *Iqaqamba kakhulu*

○ 0 ○ 1 ○ 2 ○ 3 ○ 4 ○ 5 ○ 6 ○ 7 ○ 8 ○ 9 ○ 10

39. Nceda uchaze iingxaki zempilo ezongezekileyo ezintlanu ezikuthukuthezelayo:

☐ Akukho zingxaki zempilo zongezekileyo endinazo

✎ ....

✎ ....

✎ ....

✎ ....

✎ ....

40. Nceda uphawule isimo sakutshaya:

○ Zange ndatshaya

- ○ Ndakhe ndatshaya
- ○ Ndiyatshaya ngoku (kuquka umntu otshaya ngelo xesha)

Imisetyenzana nokuthatha inxaxheba

Eli candelo lilandelayo linge ngxaki ohlangabezana nazo ebomini bakho. Nceda thathela ingqalelo iintsuku ezimbi nezintle xa ucinga.

	Kwezi veki zine zidlulileyo, kukangakanani ngokwengxaki othe wahlangabezana nayo...	1 Akukho ngxaki	2	3	4	5 Ingxaki enkulu
41.	... ukuqhubekeka nezinto zakho zosuku?	○	○	○	○	○
42.	... ukumelana noxinzelelo lwakho?	○	○	○	○	○
43.	...ukwenza izinto ezizakufuna usebenzise izandla zakho kunye neminwe, njengoku phakamisa izinto okanye ukuvula ikhonteyina?	○	○	○	○	○
44.	...ukufikelela apho ufuna ukuya khona?	○	○	○	○	○
45.	...ukusebenzisa izithuthi zikawonke-wonke?	○	○	○	○	○
46.	... ukusebenzisa isithuthi zabucala?	○	○	○	○	○
47.	...ukujongana nempilo yakho,ukutya kakuhle, ukuzilolonga okanye ukusela amayeza akho?	○	○	○	○	○
48.	...ukwenza umsebenzi wakho wasendlwini uwugqibe?	○	○	○	○	○
49.	... ukunikeza uncedo okanye inkxaso kwabanye?	○	○	○	○	○
50.	... ukunxibelelana nabanye abantu?	○	○	○	○	○
51.	... ukuthandana?	○	○	○	○	○
52.	... ukwenza izinto zokuphumla okanye ukuzonwabisa?	○	○	○	○	○
53.	... ukuqhawukelwa ngumphefumlo xa uzilolonga?	○	○	○	○	○
54.	Uyakwazi ukuhlala phantsi ungaxhaswanga?<br>○ Hayi<br>○ Ewe → Ingaba kuyingxaki kangakanani ukuhlala phantsi ixesha elide njengma-30 emizuzu?	○	○	○	○	○
55.	Uyakwazi ukuma ungaxhaswanga?	○	○	○	○	○

- ○ Hayi
- ○ Ewe → **Ingaba kuyingxaki kangakanani ukuma ixesha elide njengma-30 emizuzu?**

Le mibuzo ibuza ngokukwazi kwakho ukwenza imisetyenzana equka ukuhambahamba. Khetha impendulo echaza ngcono ukwazi ukuzenzela izinto ngaphandle kokuncedwa ngomnye umntu kodwa usebenzisa izixhobo okanye ubuxhakaxhaka obukade ubusebenzisa (umz., iibhodi zokuthwala umntu, izinyusi iibhedi zesibhedlele).

	**Uyakwazi uku...**	*Ngaphandle kobunzima*	*Kunzima kancinci*	*Kunzinyana*	*Kunzima kakhulu*	*Uwukwazi*
**56.**	**... ukuphakama emgangathweni ukusuka phantsi xa ubulele ngomqolo?**	○	○	○	○	○
**57.**	**ukutyhala ucango olunzima luvuleke?**	○	○	○	○	○
**58.**	**...ukusuka xa ubuhleli ecaleni kwebhedi ufuna ukucambalala ngomqolo?**	○	○	○	○	○

Ukuzimela kwizinto ozenzayo ngosuku lwakho

Kwinto nganye, nceda qwalasela ibhokisi emelene nenkcazelo echaza imeko yakho ngoku. Nceda ufunde okubhaliweyo ngononophelo kwaye ukrwele ibhokisi enye kwicandelo ngalinye.

59. Ukutya nokusela

- ○ Ndidinga ukutyiswa ngophayiphu abafakwa emqaleni okanye esisuswini
- ○ Ndidinga ukuncediswa xa ndisitya / ndiseka
- ○ Ndidinga ukuncediswa kancinane xa ndisitya / ndisela okanye ndifaka / ndikhulula izixhobo zokuncedisa
- ○ Ndiyazityela / ndiyaziselela ngokwam, kodwa ndidinga izixhobo ezincedisa okanye uncedo ukusika ukutya, ukugalela isiselo okanye ukuvula izigcini kutya.
- ○ Ndiyazityela / ndiyaziselela ngokwam ngaphandle kokuncediswa okanye izixhobo zokuncedisa

60. Ukuhlamba amantla omzimba nentloko

Oku kuquka ukufaka isephu nokosula, nokusetyenziswa amanzi etepu.

- ○ Ndidinga ukuncediswa kangangoko
- ○ Ndidinga ukuncediwa kancinane
- ○ Ndiyazenzela kodwa ndidinga izixhobo ezincedisayo okanye izixhobo ezikhethekileyo (umz., izibonda, izitulo)
- ○ Ndiyazenzela kwaye andidingi zixhobo zincedisayo okanye izixhobo ezikhethekileyo

61. Ukuhlamba umzimba ongezantsi

Oku kuquka ukufaka isephu nokosula, nokusetyenziswa amanzi etepu.

- ○ Ndidinga ukuncediswa kangangoko
- ○ Ndidinga ukuncediswa kancinane
- ○ Ndiyazenzela kodwa ndidinga izixhobo ezincedisayo okanye izixhobo ezikhethekileyo (umz., izibonda, izitulo)
- ○ Ndiyazenzela kwaye andidingi zixhobo zincedisayo okanye izixhobo ezikhethekileyo

62. Ukunxiba impahla kumzimba ongentla

Oku kuquka ukunxiba nokukhulula iimpahla ezinjengezikipa, iiblawuzi, iihempe, iibhodi, iholi, okanye izixhasi-mzimba (umz. isixhasi-ngalo, isixhasi-ntamo, ikhosethi).

- *Iimpahla ezinxibeka lula zezo zingenamaqhosha, ziziphu okanye iileyisi*
- *Iimpahla ezinxibeka nzima zinamaqhosha, iziziphu okanye iileyisi*

- ○ Ndidinga ukuncediswa kangangoko
- ○ Ndidinga ukuncediswa kancinane, nokuba ziimpahla ekulula ukunxiba
- ○ Andidingi kuncediswa ngeempahla ekulula ukunxiba, kodwa ndidinga izixhobo ezincedayo okanye ezikhethekileyo
- ○ Ndiyakwazi ukuzinxibela iimpahla ekulula ukuzinxiba kwaye ndidinga nje uncedo okanye izixhobo zoncedo okanye iimeko ezikhethekileyo xa ndinxiba iimpahla elinzima ukuyinxiba
- ○ Ndiyazinxibela ngokupheleleyo

63. Ukunxiba umzimba ongezantsi

Oku kuquka ukunxiba nokukhulula iimpahla ezinjengooshoti, iibhulukhwe, iikawusi, iibhanti okanye izixhasi-mzimba (umz. isixhasi-mlenze).

- *Iimpahla ezinxibeka lula zezo zingenamaqhosha, ziziphu okanye iileyisi*
- *Iimpahla ezinxibeka nzima zinamaqhosha, iziziphu okanye iileyisi*

- ○ Ndidinga ukuncediswa kangangoko
- ○ Ndidinga ukuncediswa kancinane, nokuba ziimpahla ekulula ukunxiba
- ○ Andidingi kuncediswa ngeempahla ekulula ukunxiba, kodwa ndidinga izixhobo ezincedayo okanye ezikhethekileyo
- ○ Ndiyakwazi ukuzinxibela iimpahla ekulula ukuzinxiba kwaye ndidinga nje uncedo okanye izixhobo zoncedo okanye iimeko ezikhethekileyo xa ndinxiba iimpahla elinzima ukuyinxiba

- Ndiyazinxibela ngokupheleleyo

64. Ukuzicoca

Umz., imisetyenzana enjengokuhlamba izandla nobuso, ukuxukuxa, ukukama, ukusheva, okanye ukuthambisa.

- Ndidinga uncedo kangangoko
- Ndidinga uncedo kancinane
- Ndiyazenzela xa kukho izixhobo zokuncedisa
- Ndiyazenzela ngaphandle kwezixhobo zokuncedisa

65. Ukulawula isinyi

Nceda ucinge ngendlela okhupha ngayo umchamo kwisinyi.

A. Ukusetyenziswa kwekhathithha efakwe ngaphakathi

- Ewe → ***Nceda uye kumbuzo wama-66***
- Hayi → *Nceda uphendule u-B no-C.*

B. Ikhathitha yesiqabu

- Ndidinga uncediso kangangoko
- Ndiyenza ngokwam kodwa ndincediswa (ukuzifaka ikhathitha)
- Ndiyenza ngokwam kungekho luncedo (ukuzifaka ikhathitha)
- Andiyisebenzisi

C. Ukusetyenziswa kwesixhobo sokudontsa sangaphandle *.(umz. ikhathitha yekhondom, iinapkeni)*

- Ndidinga uncedo kangangoko ukuwasebenzisa
- Ndidinga uncedo kancinane ukuwasebenzisa
- Ndiwasebenzi ngaphandle koncediso
- Ndiyawababamba umchamo kwaye andisebenzisi zixhobo zokudontsa zangaphandle

66. Ukulawula ukuzithuma

A. Ingaba udinga uncedo kulawulo lokuzithuma *(umz. ukufaka amayeza ngaphantsi)***?**

- Ewe
- Hayi

B. Ukuzithuma kwam…

- akwenzeki rhoqo okanye kuhlale kuhlale kwenzeke (ngaphantsi kwesinye ngeentsuku ezi-3)
- rhoqo (kanye neentsuku ezi-3 okanye ngaphezulu)

C. Ukuzithuma okungalawulekileyo ("iingozi") kwenzeka …

- Ntsuku zonke
- Kanye ukuya kwisithandathu ngeveki
- Kanye ukuya kwisine ngenyanga
- ngaphantsi kwesinye ngenyanga
- Zange kwenzeka

67. Ukusebenzisa ithoyilethi

Nceda ucinge ngokusebenzisa ithoyilethi, ukuhlamba kummandla wangaphantsi nezandla, ukunxiba nokukhulula impahla, nokusebenzisa amanapkeni.

- Ndidinga uncedo kancinane kwaye andikwazi ukuzicoca ngokwam
- Ndidinga uncedo kancinane kodwa ndikwazi ukuzicoca ngokwam
- Andidingi luncedi kodwa ndidinga izixhobo zoncediso (umz. izibonda) okanye imeko ekhethekileyo (umz. isitulo esifikelelayo ethoyilethi)
- Andidingo naluphi na uncedo, izixhobo zokuncedisa okanye imeko ekhethekileyo

68. Yeyiphi kule misetyenzana ilandelayo ongakwazi ukuyenza ngaphandle kokuncediswa okanye izincedisi zombane?

Krwela konke okungasebenza

- ☐ Ukuguqula umzimba wakho ongentla xa usebhedini
- ☐ Ukuguqula umzimba wakho ongentla xa usebhedini
- ☐ Ukuhlala ebhedini
- ☐ Ukuzinyusa uhleli esitulweni okanye kwisitulo esinamavili
- ☐ Akukho, ndidinga ukuncediswa kuyo yonke lemisetyenzana

69. Ukusuka ebhedini ukuya esitulweni esihambayo

- ○ Ndidinga uncedo kangangoko
- ○ Ndidinga uncediso kancinane, ukunakekelwa okanye izixhobo zokuncedisa (umz. ibhodi etshibilizayo)
- ○ Andidingi naluphi na uncedo okanye izixhobo ezincedisayo
- ○ Andidingi kusebenzisa isitulo esihambayo

70. Ukuhambahamba imigama emifutshane (iimitha ezi-10 ukuya kwi-100)

Ndisebenzisa isitulo esihambayo. Ukuhambahamba, ...

- ○ Ndidinga uncediso kangangoko
- ○ Ndidinga isitulo esizihambelayo sombane okanye uncediso oluncinane ukusebenzisa isitulo esihambayo
- ○ Ndiyazenzela yonke into kwisitulo esihambayo

Ndihamba imiganyana ephakathi kwaye...

- ○ ndidinga unakekelo ngelixa ndihambayo (kukho okanye kungekho zincedisi zokuhamba)
- ○ ndihamba ngesakhelo sokuhamba okanye iintonga zokuhamba, ndijula imilenze yomibini ukuyisa phambili ngexesha
- ○ ndihamba ngeentonga okanye ikheyini ezimbini, ndibeka unyawo olunye phambi kolunye
- ○ ndihamba ngekheyini enye
- ○ ndihamba ngesixhasi-mlenze kuphela (umz. izixhasi-mlenze)
- ○ ndihamba ngaphandle kwezincedisi

71. Ingaba belisithini igama okanye isikhundla somsebenzi wakho obungundoqo ngaphambi komonzakalo kumnqonqo?

- ○ Bendingenamsebenzi ngaphambi komonzakalo kumnqonqo.
- ○ Igama okanye isikhundla somsebenzi wam ongundoqo ibi (nceda uchaze ngqo kangangoko, umz. ungathi u'mabhalana' kodwa uthi 'umabhalana ebhankini', ungathi u'mphathi' nje kodwa yithi 'umphathi weentengisi'):

✐ ....

72. Ingaba uthe wafumana iinkonzo zovuselelo <u>ngokomsebenzi</u> emva komonzakalo womnqonqo?

umz. iingcebiso ngezomsebenzi, uqeqesho kwakhona kwezomsebenzi, uqeqesho kwizakhono zomsebenzi

- ○ Ewe
- ○ Hayi

73. Emva kokuba ukhutshiwe kwicandelo labavuselelwa bengaphakathi lokuqala, ingaba ikuthabathe ixesha elingakanani ngaphambi kokuba uqale or ubuyele kumsebenzi ohlawulwayo?

- ○ Andizange ndasebenza emva kokuvuselelwa kwangaphakathi kokuqala
- ○ Nje emva kokuvuselelwa kwangaphakathi kokuqala
- ○ Ndibuyele emsebenzini emva kweminyaka e ✐ .... neenyanga ezi ✐ ....

74. Ingaba ufumana ipenshini yokonzakala okanye esinye nje isibonelelo somonzakalo?

- ○ Ewe
- ○ Hayi

75. Ingaba ithini imeko yakho yokusebenza ngoku?

Krwela konke okungasebenza

- ☐ Ndisebenzela umvuzo kumqeshi iiyure ezi ✐ .... ngeveki
- ☐ Ndisebenzela umvuzo kumqeshi iiyure ezi ✐ .... ngeveki, kodwa ngoku ndikwikhefu lokugula ngaphezu kweenyanga ezintathu
- ☐ Ndiyazisebenzela, ndisebenza iiyure ezi ✐ .... ngeveki
- ☐ Ndisebenza njengelungu losapho elingahlawulwayo (umz. ukusebenza kwishishini losapho)
- ☐ Umfazi ogcina ikhaya / indoda egcina ikhaya
- ☐ Umfundi
- ☐ Andiphangeli
- ☐ Ndidla umhlalaphantsi ngenxa yokugula
- ☐ Ndidla umhlalaphantsi ngenxa yobudala
- ☐ Enye, nceda uchaze: ✐ ....

76. Ingaba wenza umsebenzi <u>ohlawulayo</u>?

- ○ Ewe
- ○ Hayi → *Nceda ugqithele kumbuzo wama-84*

77. Ingaba lithini igama okanye isikhundla somsebenzi wakho ongundoqo?

Nceda ucacise kangangoko, umz. ungathi u'mabhalana' kodwa uthi 'umabhalana ebhankini', ungathi u'mphathi' nje kodwa yithi 'umphathi weentengisi':

✐ ....

78. Ingaba ufna ukusebenza ngaphezulu, ngaphantsi okanye isixa seeyure ezilinganayo nezo ukuzisebenza ngaphambili?

- ○ Iiyure ezingaphezulu

- ○ Iiyure ezingaphantsi
- ○ Isixa esifanayo

	1 Akukho ngxaki	2	3	4	5 Kukho ingxaki enkulu
**79. Ingaba kuyingxaki engakanani ukuba wenze izinto ziqhube njengoko zifunwa emsebenzini?**	○	○	○	○	○
**80. Ingaba kuyingxaki kangakanani ukufikelela emsebenzini?** *Umz. ukufikelela kwisakhiwo, iofisi okanye ithoyilethi yakho*	○	○	○	○	○

	Kakhulu	Kwinxalenye enkulu	Kwiinxalenye	Kancinane	Andiyidingi tu kwaphela	Andinasidingo sinjalo
**81. Ingaba unazo izixhobo zokukuncedisa ongasisebenzisa xa usemsebenzini ?** *umz., izixhobo ezincedisayo zekhompyutha, iitafile ezilungelelaniswayo okanye izixhasi-ngalo /izandleokanye izixhasi-milenze.*	○	○	○	○	○	○

Le mibuzo mibini ilandelayo ibhekisa kumsebenzi wakho kwangoku. Kwintetha nganye kwezi zilandelayo, nceda uphawule ukuba ingaba uyavuma kakhulu, uyavuma, awuvumi okanye awuvumi kakhulu.

	Uvuma kakhulu	Uyavuma	Awuvumi	Awuvumi kakhulu
**82. Ndifumana ukwamkeleka okundifaneleyo emsebenzi wam.**	○	○	○	○
**83. Xa ndiqwalasela zonke iinzame zam kunye nendikufezekisileyo, umvuzo wam awanelanga.** *→ Nceda uye kumbuzo wama-87*	○	○	○	○

84. Ingaba uthanda ukuba nomsebenzi ohlawulwayo?

- ○ Ewe
- ○ Hayi

85. Ingaba uziva ukulungele ukwenza umsebenzi ohlawulayo?

- ○ Ewe, iyure e-1 ukuya kwezi-11 ngeveki
- ○ Ewe, iiyure ezi-12 ukuya kwezingama-20 ngeveki
- ○ Ewe, iiyure ezingaphezu kwama-20 ngeveki
- ○ Hayi, andifuni tu kwaphela

86. **Zithini izizathu ezibangela ukuba ube awusebenzi ngoku?**
Krwela oko kuhambelanayo

- ☐ Imeko zempilo okanye zokukhubazeka
- ☐ Ndisafuunda okanye ndisaqeqeshwa
- ☐ Uxanduva losapho
- ☐ Andiwufumani umsebenzi ondifaneleyo
- ☐ Andiyazi ukuba ndiwufune njani okanye ndiwukhangele njani umsebenzi
- ☐ Andinazidingo zezimali
- ☐ Abazali okanye iqabane alifuni ukuba ndisebenze
- ☐ Iinkonzo zothutho ezinqongopheleyo
- ☐ Ukungafikeleli kwiindawo ezinganengqesho (umz., ukungena kwizakhiwo, iofisi okanye ithoyilethi yakho)
- ☐ Ukunqongophala kwezixhobo ezincedisayo
- ☐ Ukoyika ukulahlekelwa sisibonelelo sokukhubazeka (umz., iintlawulo zepenshini, ikhava yeinshorensi yempilo)
- ☐ Andifuni kusebenza
- ☐ Okunye, nceda ucacise: ✎ ……………………………………………………………………………..

Iimeko zendalo ezisingqongileyo

Kubomi bethu bemihla ngemihla, siba kwimpembelelo zangaphandle zezinto ezahlukeneyo okanye iimeko zendalo ezisingqongileyo. Ezi zinto zingenza ubomi bemihla ngemihla bube lula okanye bube nzima. Cinga ngezi veki zine zidlulileyo, nceda uthekelelele ukuba ingaba ezi meko zendalo ezisingqongileyo zinefuthe elingakanani kwintatho-nxaxheba yakho phakathi koluntu.

	Ayingeni	*Ayinafuthe*	*Yenza ubomi bam bube nzinyana*	*Yenza ubomi bam buze nzima*
**87.** **Ukungafikeleli okanye ukungakwazi ukufikelela kwiindawo zoluntu**<br>*Umz., ukungafikeleleki kwezakhiwo zoluntu, iipaki*	○	○	○	○
**88.** **Ukungafikeleli okanye ukungakwazi ukufikelela kumakhaya abahlobo nezalamane**	○	○	○	○
**89.** **Iimo zezulu ezingentlanga**<br>*Umz., imozulu, ixesha lonyaka, amaqondo obushushu, ulophu*	○	○	○	○
**90.** **Izimvo zoluntu ezingentlanga ngakubantu abakhubazekileyo**<br>*umz., ukucalula, ityheneba, ukungahoyi*	○	○	○	○
**91.** **Izimvo ezingentlanga zosapho nezalamane malunga nokukhubazeka kwakho**<br>*umz., ukucalula, ukunqongophala kwenkxaso, imeko yokukhuselo olugqithisileyo*	○	○	○	○
**92.** **Izimvo ezingentlanga zabahlobo bakho malunga nokukhubazeka kwakho**<br>*umz., ukucalula, ukunqongophala kwenkxaso, imeko yokukhuselo olugqithisileyo*	○	○	○	○
**93.** **Izimvo ezingentlanga zabamelwane, abantu obaziyo noogxa bakho emsebenzini malunga nokukhubazeka kwakho**<br>*umz., ukucalula, ukunqongophala kwenkxaso, imeko yokukhuselo olugqithisileyo*	○	○	○	○

94.	**Ukunqongophala okanye ukungabikho kobungcaphephe kwezincedisi ezizakwenza ukwazi ukuhambahamba imiganyana emifutshane**<br>*Umz. Izitepusi ezihamba ngombane, ikheji, izincedisi-kuhamba okanye isitulo esinamavili*	○	○	○	○
95.	**Ukunqongophala okanye ukungafaneleki kwezinto zothutho kwimigama emide**<br>*Umz. ukunqongophala kweemoto ezifanelekileyo okanye kunzima ukusebenzisa izithuthi zikawonke wonke*	○	○	○	○
96.	**Ukunqongophala okanye ukunganeliseki ngoncedo lwamanesi kunye neenkonzo zenkxaso**<br>*Umz. Uncedo lwezempilo ekhaya okanye ukuncediswa wena buqu.*	○	○	○	○
97.	**Ukunqongophala okanye ukungoneliswa ngamayeza kunye nezixhobo nezibonelelo zonyango**<br>*Umz., umbhobho womchamo, izibulali-ntsholongwane, izixhasi, imiqamelelo*	○	○	○	○
98.	**Iimeko zeengxaki zezimali**<br>*Umz., ukunqongophala kwemali*	○	○	○	○
99.	**Ukunqongophala okanye ukungoneli kwezixhobo zokunxibelelwano**<br>*Umz., ukunqongophala okanye ukunganeli kwezixhobo zokubhala, iikhompyutha, ifowuni, iimawusi*	○	○	○	○
100.	**Ukunqongophala okanye ukungoneli kweenkonzo zikarhulumente**<br>*umz., impepha ezixhasa ukukhubazeka okanye ezinye izibonelelo*	○	○	○	○

Inkonzo zempilo

101. Ngobani ababoneleli ngononophelo lwempilo othe wabandwendwela okanye ngoobani abathe bakundwendwela ekhaya kwezi <u>nyanga zilishumi elinambini zidlulileyo?</u>

Krwela konke okungenayo

- ☐ Ugqirha osekuhlaleni/ugqirha okwinkonzo zempilo ekuhlaleni/ugqirha wokukubuyisela kwisimo sakho/ugqirha oyingcaphephe kumonzakalo womnqonqo
- ☐ Enye ingcaphephe yogqirha *umz., ugqirha wotyando, ugqirha wabafazi, ugqirha wengqondo, ugqirha wamehlo*
- ☐ Umongikazi okanye umbelekisi
- ☐ Ugqirha wamazinyo
- ☐ Umeluli wamathambo
- ☐ Ingcali yamathambo
- ☐ Umncedisi wezandla nokwenza umsebenzi
- ☐ Igcisa lokusebenza ngengqondo
- ☐ Umntu onyanga ngezinye iindlela zonyanga *umz.,umntu osebenzisa amayeza esintu, umntu onyanga ngeenaliti*
- ☐ Usomachiza
- ☐ Unompilo emakhayeni
- ☐ Abanye, nceda ucacise: ✎ ....
- ☐ Andikhange ndindwendwele nawuphi na umboneleli ngeenkonzo zonakekelo lwempilo kwezi nyanga zilishumi elinambini zidlulileyo

102. Kwezi nyanga zilishumi linambini zidlulileyo, zingaphi izihlandlo oye wangeniswa njengesigulane esibhedlele, izakhiwo zovuselelo okanye ezinye izakhiwo zonakekelo kangangenyanga ubuncinane?

✎ …………… *(izihlandlo)*

	Undwendwelo lwakho kumboneleli ngonakekelo lwempilo, ungazithelekela njani ezi meko zilandelayo:	*Lunge kakhulu*	*Lungile*	*Lungaluhlanga kodwa lungelubi*	*Lubi*	*Lubi kakhulu*
**103.**	**...amava akho ngokuphathwa ngentlonipho?**	○	○	○	○	○
**104.**	**…ababoneleli ngonakekelo lwempilo bazichaze njani izinto kuwe?**	○	○	○	○	○
**105.**	**…amava akho ekwenziweni kwezigqibo ngonyango lwakho?**	○	○	○	○	○

106. Kwezi nyanga zilishumi elinambini zidlulileyo, ukhe wadinga unakekelo lwempilo kodwa alufumana?

- ○ Hayi
- ○ Ewe. Zeziphi izizathu ezicacusa kutheni ungakwazanga ukufumana unakekelo lwempilo oludingayo?

  Krwela konke oko kuhambelanayo

  - ☐ Andikhange ndikwazi ukumelana neendleko zotyelo
  - ☐ Bekungekho zinkonzo
  - ☐ Akukho zithuthi zikhoyo
  - ☐ Andikhange ndikwazi ukumelana neendleko zezothutho
  - ☐ Ndandiphethwe kakubi kwixa elidlulileyo
  - ☐ Bendingakwazi ukuphuma emsebenzini okanye bekukho ezinye izinto ezindibambileyo
  - ☐ Amachiza okanye izixhobo zomboneleli ngonakekelo lwempilo bezinganelanga
  - ☐ Izakhono zomboneleli ngonakekelo lwempilo bezinganelanga
  - ☐ Andazanga ukuba mandiyephi
  - ☐ Uzamile kodwa walelwa unakekelo lwempilo
  - ☐ Ucinge ukuba awuguli
  - ☐ Okunye, nceda ucacise: ✎ ……………………………………………………………………………

		Ndanelisele kakhulu	*Ndanelisekile*	*Ndaneliseke ndinganelisekanga*	*Andanelisekanga*	*Andanelisekanga tu kwaphela*
**107.**	**Ngokuphangaleleyo, ingaba waneliseke kangakanani ngeenkonzo zempilo eziqhutywa kummandla wakho?**	○	○	○	○	○

Iimeko zobuqo

Le mibuzo ilandelayo ingokuba usibona njani isiqu sakho.

	1 Andizithembanga tu	2	3	4	5 Ndizithembe kakhulu
**108. Uzithembe kangakanani ukuba ube ungafumana iindlela zokufumana loo nto uyifumana ukuba kukho umntu okuphikisayo?**	○	○	○	○	○
**109. Uzithembe kangakanani ukuba ungajongana ngqo neziganeko ezingalindelekanga?**	○	○	○	○	○
**110. Uzithembe kangakanani ukuba ube ungagcina uqhagamshelwano nabantu ababalulekileyo kuwe?**	○	○	○	○	○
**111. Uzithembe kangakanani ukuba ungazigcina ukwimpilo entle?**	○	○	○	○	○
**112. Ingaba ucinga ukuba ukuphila nomonzakalo komnqonqo kukwenze wangumntu owomeleleyo?**	○	○	○	○	○
**113. Ingaba unenkxalabo yokuba kuza kwenzeka ntoni kwixa elizayo?** *Umz., cinga ngokungakwazi ukuzinakekela, okanye ukuba ngumthwalo kwabanye kwixesha elizayo*	○	○	○	○	○
**114. Ingaba ucinga ukuba uza kukwazi ukufezekisa amaphupha, amathemba, neminqweno yakho?**	○	○	○	○	○
**115. Ingaba ukhe wenze izigqibo ezinkulu ngobomi bakho?** *Umz. ukugqiba apho uza kuhlala khona okanye ingaba uza kuhlala nabani, uza kuyichitha njani imali yakho*	○	○	○	○	○
**116. Ingaba uziva ubandakanyeka xa uphakathi kwabanye abantu?**	○	○	○	○	○

117. Ingaba kwezi nyanga zilishumi elinambini zidlulileyo, kukhe wehlelwa sisiganeko esibi esikhulu ebomini bakho?

Umz. imeko exhalabisayo yempilo okanye ingozi, ukuxabana nabanye abantu, ukuqhawula umtshato okanye ukuswelekelwa ngomntu omthandayo

○ Hayi

○ Ewe, nceda ucacise: ✎ ....

Ikhwaliti yobomi nempilo ngokubanzi

Le mibuzo ilandelayo ingokuba uyithelekelela njani ikhwaliti yobomi bakho kwezi ntsuku zilishumi elinesine zidlulileyo. Nceda ucinge ngamanqanaba, amathemba, iziyolo neenkxalabo.

Kwezi ntsuku zilishumi elinesine zidlulileyo ...	*Iphantsi kakhulu*	*Iphantsi*	*Ayikho phantsi kodwa ayikho phezulu*	*Iphezulu*	*Iphezulu kakhulu*
**118. Ingaba ungayithelekelela kowuphi umyinge ikhwaliti yobomi bakho?**	○	○	○	○	○

	Andanelisekanga kakhulu	*Andanelisekanga*	*Ndaneliseke ndinganelisekanga*	*Ndanelisekile*	*Ndaneliseke kakhulu*
**119. Ingaba waneliseke kangakanani ngempilo yakho?**	○	○	○	○	○
**120. Ingaba waneliseke kangakanani ngokwenza imisetyenzana yemihla ngemihla?**	○	○	○	○	○
**121. Ingaba waneliseke kangakanani ngesiqu sakho?**	○	○	○	○	○
**122. Ingaba waneliseke kangakanani ngobudlelwane bakho nabanye abantu?**	○	○	○	○	○
**123. Ingaba waneliseke kangakanani ngeemeko zakho zokuphila?**	○	○	○	○	○

124. Ngokuphangaleleyo, ungathi impilo yakho:

○ Ibalasele
○ Ilunge kakhulu
○ Ilungile
○ Iphakathi nje
○ Ihluphekile

125. Xa uthelekisa sithuba sonyaka odlulileyo, ingaba ungayithelekela njani impilo yakho ngokuphangaleleyo ngoku?

○ Ingcono kakhulu
○ Ingconwanyana
○ Iyafana
○ Iyehla
○ Yehle kakhulu

Enkosi ngokuthabatha inxaxheba kuvavanyo-zimvo lwe-InSCI!

International Spinal Cord Injury Survey (InSCI)

Afrikaanse weergawe

Die eerste wereldwye opname van persone met spinale koord beserings wat in die gemeenskap woon.

Lande regoor die wereld neem deel aan hierdie initiatief wat behels die evaluasie van die geleefde ervaring om met 'n spinale koord besering te lewe deur diegene te vra wie die beste weet: persone met spinale koord beserings

In samewerking met

ISPRM International Society of Physical and Rehabilitation Medicine

World Health Organization

ISCOS The International Spinal Cord Society

Beste Deelnemer

Welkom by die InSCI opname, ons is baie gelukkig om u aan boord te hê!

InSCI is die eerste wêreldwye opname oor gemeenskapsgebaseerde woning van persone met rugmurg beserings.Lande regoor die wêreld neem deel aan hierdie initiatief om vas te stel hoe dit is om saam met 'n spinale koord besering te lewe deur die te vra wat die beste kennis het: persone met spinale koord beserings.

Kan u asseblief die vraelys in vul so deeglik as moontlik en moet nie vrae ontgeantwoord laat nie. Daar is geen regte of verkeerde en geen goeie of slegte antwoord nie. Dit is belangrik dat u die spontaan antwoord en self besluit watter opsie die best van toepassing is tot u persoonlike situasie.

U kan ook die vraelys elektronies beantwoord by [www.insci.com]. Meld asseblief aan met u InSCI-ID en persoonlike wagwoord:

U InSCI-ID is: ######
U se persoonlike wagwoord is: ######

Ons waarborg dat u data beskerm is met die hoogste sekuriteit standaarde. Geen persoonlike data sal oorgehandel word aan 'n derde persoon wat nie deel van die studie sentrum is nie. All vraelyste is anoniem en word by 'n unieke nommer (InSCI-ID) herken, en daar is geen persoonlike informasie soos name of adres op die papier of elektroniese vraelys nie.

In die geval u enige vrae of hulp nodig het met die vraelys, skakel ons gerus. Stuur ons asseblief 'n e-pos aan contact@rsi.insci.network of skakel ons tolvry InSCI-helplyn by 021 959 2542.

Weereens dankie vir u verbintenis!

U InSCI-Span

Dr Conran Joseph

Persoonlike Inligting

1. Dui asseblief u geslag aan:

○ Manlik
○ Vroulik

2. Op watter dag, maand en jaargetal was u gebore?

DD / MM / JJJJ

□□/□□/□□□□

3. In watter land was u gebore?

…………………………………………..…………

4. Wat is u huidige huwelikstaat?

○ Enkellopend
○ Getroud
○ Saamwoonverhouding of in vennootskap
○ Uiteengegaan of geskei
○ Weduwee of wewenaar

5. Wie maak nog deel uit van u huishouding?

Merk als wat van toepassing is

□ Ek woon alleen
□ Kinders onder 14 jaar, aantal: ……………
□ Jeug tussen 14 en 18 jaar oud, aantal: ……………
□ Persone tussen 18 en 64 jaar oud, aantal: ……………
□ Persone ouer as 64 jaar oud, aantal: ……………
□ Ek woon in 'n instelling b.v. *ouetehuis, verpleeginrigting……..*

6. Kry u bystand vir u dag-tot-dag aktiwiteite by die huis of buite?

○ Nee
○ Ja, by die volgende persone:

Merk als wat van toepassing is

□ Familie
□ Vriende
□ Professionele of betaalde helpers

7. Wat is die hoogste vlak van opvoeding wat u voltooi het?

○ Primêre
○ Laer sekondêre
○ Hoër sekondêre
○ Verkorte tersiêre
○ Baccalaureus Graad of ekwivalent
○ Meesters of ekwivalent
○ Ander, naamlik: ……………………………………………………

8. **Heoveel jare van studie het u voltooi?**

Jare van opvoeding en opleiding voor die spinale koord besering: .... *(aantal jare)*

Jare van opvoeding en opleiding na die spinale koord besering: .... *(aantal jare)*

9. **As u in ag sou neem al die werkende persone in u huishouding wat 'n salaries of loon verdien, wat is die totale inkomste [na belasting] van u huishouding per maand?**

- ○ Minder as R1100 per maand
- ○ R1101 – R3000 per maand
- ○ R3001 –R4500 per maand
- ○ R4501 – R6000 per maand
- ○ R6001 –R9000 per maand
- ○ R9001 – R12000 per maand
- ○ R12001 – R20 000 per maand
- ○ R20001 – R3000 per maand
- ○ R30001 – R50000 per maand
- ○ R500001 of meer

10. **Dink aan hierdie leer as verteenwoordigend aan waar mense staan in Suid-Afrika.**

Aan die bo-punt van die leer is die mense wat die beste daaraan toe is – diegene wat die meeste geld besit, die hoogste geleerdheid asook die mees gerespekteerde beroepe. Aan die onderste punt van die leer is diegene wat die minste geld het, die minste geleerdheid asook die minste gerespekteerde beroepe en ook geen werk nie. Hoe hoer op die leer u uself bevind, hoe nader is u aan die persone aan die toppunt, en hoe laer op die leer u uself bevind, hoe nader is u aan die persone op die laagste punt.

Waar sal u uself op hierdie leer plaas?

Plaas asseblief 'n groot X op die rang waar u dink u staan op hierdie tydstip van u lewe, in verhouding tot ander mense in Suid-Afrika.

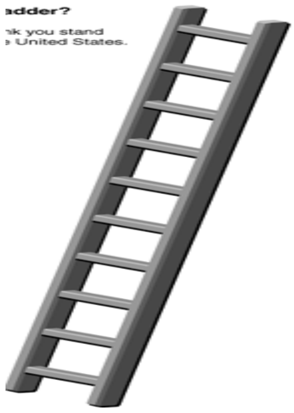

11. **Beskryf asseblief die vlak van u spinale koord besering.**

- ○ Parapleeg (normale krag in arms, hande en vingers)
- ○ Tetrapleeg (Geen of abnormale beweging or gevoel in arms en bene)

12. **Is u besering volledig (complete) of onvolledig (incomplete)?**

- ○ Volledig (geen gevoel in enige deel van die liggaam onder die beseringsvlak).
- ○ Onvolledig (het gevoel en kan 'n deel of dele van die liggaam beweeg onder beseringsvlak.

13. **Dui asseblief die oorsaak van u spinale koord besering aan:**

Oorsaak deur besering:

Merk als wat van toepassing is

Bv. As u ongeluk gedurende werk merk, moet u ook aandei of dit 'n val of ander oorsaak van besering was.

- ☐ Ongeluk gedurende sport
- ☐ Ongeluk gedurende onspanningsaktiwiteite
- ☐ Ongeluk gedurende werk
- ☐ Verkeersongeluk
- ☐ Besering as gevolg van geweld bv. *skietwond*
- ☐ 'n Val van minder as 1 meter
- ☐ 'n Val van meer as 1 meter
- ☐ Ander oorsaak van besering: ....

Oorsaak a.g.v. siekte:

Merk als wat van toepassing is

- ☐ Degenerasie van die spinalekolom
- ☐ Gewas - Goedaardig
- ☐ Gewas – kwaadaardig (kanker)
- ☐ Vaskulêre problem (bv.bloedloosheid, bloedvloeiing, misvorming)
- ☐ Infeksie (bv. Bakterieel, virus)
- ☐ Ander: ....

14. **Dui asseblief so presies as moontlik die datum aan waarop die spinale koordbesering plaasgevind het.**

DD / MM / JJJJ

☐☐/☐☐/☐☐☐☐

Energie en gevoelens

Hierdie vrae gaan oor hoe u voel en hoe dit met u die laaste 4 weke gesteld was. Gee vir elke vraag die een antwoord wat die naaste beskryf hoe u gevoel het.

**Hoeveel van die tyd gedurende die laaste 4 weke**		*Al die tyd*	*Meeste van die tyd*	*Sommige tye*	*Baie min*	*Nooit nie*
15.	Het u lewenslustig gevoel?	○	○	○	○	○
16.	Was u baie senuweeagtig?	○	○	○	○	○
17.	Was u so terneergedruk dat niks vir u wou werk nie?	○	○	○	○	○
18.	Het u kalm en rustig gevoel?	○	○	○	○	○
19.	Was u energiek?	○	○	○	○	○
20.	Was u terneergedruk en depressief?	○	○	○	○	○
21.	Het u afgemat gevoel?	○	○	○	○	○
22.	Was u gelukkig?	○	○	○	○	○
23.	Was u moeg?	○	○	○	○	○

Gesondheidsprobleme

Beoordeel asseblief in hoe 'n mate die volgende gesondheidsprobleme die laaste 3 maande vir u probleme besorg het. As u die bepaalde gesondheidsprobleem ondervind het, dui ook aan of u behandeling daarvoor ontvang het, of nie (byvoorbeeld, medikasie ontvang of behandeling ontvang van dokter of ander gesondheidsprofessioneel).

		1 Geen probleem	*2*	*3*	*4*	*5 Uiterste probleem*	*Het u behandeling daarvoor gekry?*
**24.**	**Slaapprobleme**<br>Bv.*Dit sluit in problem om aan die slaap te raak, om deurnag te slaap en om vroeg wakker te raak..*	○	○	○	○	○	○ Ja<br>○ Nee
**25.**	**Probleme met ontlasting**<br>*Bv. Dit sluit in diarree,stoelgang onbeheerstheid (ongelukke) en konstipasie.*	○	○	○	○	○	○ Ja<br>○ Nee
**26.**	**Urinekanaalinfeksie**<br>*Bv.Dit sluit in nier- en blaasinfeksies.*	○	○	○	○	○	○ Ja<br>○ Nee
**27.**	**Blaasdisfunksie**<br>*Bv.Dit sluit in swak van blaas of nierstene, urinelekkasie, terugtrek van urine.*	○	○	○	○	○	○ Ja<br>○ Nee
**28.**	**Seksuele disfunksie**<br>*Bv. Dit sluit in disfunksie in seksuele opwekking, -ereksie en bereiking van orgasme.*	○	○	○	○	○	○ Ja<br>○ Nee
**29.**	**Kontrakture**<br>*Dit is die limitasie (tekortkoming) rakende die reikwydte van die beweging van spiere.*	○	○	○	○	○	○ Ja<br>○ Nee
**30.**	**Spiersametrekkings, spastisiteit**<br>*Dit verwys na onbeheerste, rukkerige spierbewegings, soos bv. onbeheerste spiertrekkings en krampe.*	○	○	○	○	○	○ Ja<br>○ Nee
**31.**	**Druksere ,Bedsere**<br>*Hierdie ontwikkel as 'n veluitslag of rooiheid van die vel en ontwikkel verder as 'n geïnfekteerde seer.*	○	○	○	○	○	○ Ja<br>○ Nee
**32.**	**Respiratoriese Probleme (Asemhalingsprobleme)**<br>*Simptome van respiratoriese infeksies of –probleme sluit in moeilikheid met asemhaling en toenemende uitskeidings.*	○	○	○	○	○	○ Ja<br>○ Nee
**33.**	**Beserings veroorsaak deur die gebrek aan sensasie**<br>*Bv. Dit sluit in brandwonde opgedoen deur warm vloeistof in die skoot te dra of deur te na aan die vuur of die verwarmer te sit.*	○	○	○	○	○	○ Ja<br>○ Nee
**34.**	**Sirkulasieprobleme**<br>*Dit sluit in geswelde are van voete, bene en hande, of die ontwikkeling van bloedklonte.*	○	○	○	○	○	○ Ja<br>○ Nee

	1 Geen probleem	2	3	4	5 Uiterste probleem	Het u behandeling daarvoor gekry?
**35. Outonomiese disrefleksia** *Simptome is skielike styging in bloeddruk en sweet, vlekke of puisies op die vel, pupiluitsetting en hoofpyn.*	○	○	○	○	○	○ Ja ○ Nee
**36. Posturale lae bloeddruk** *Dit veroorsaak 'n sterk sensasie van lighoofdigheid na 'n verandering van posisie as gevolg van 'n skielike daling in die bloeddruk.*	○	○	○	○	○	○ Ja ○ Nee
**37. Pyn** *Om pyn in jou daaglikse lewe te ondervind*	○	○	○	○	○	○ Ja ○ Nee

38. Beoordeel asseblief u pynvlak deur die nommer wat u pyn die beste beskryf die laaste week, te omsirkel.

Geen pyn ← → *Die ergste pyn wat u u kan voorstel*

○ 0 ○ 1 ○ 2 ○ 3 ○ 4 ○ 5 ○ 6 ○ 7 ○ 8 ○ 9 ○ 10

39. Noem asseblief vyf addisionele gesondheidsprobleme wat u verder pla:

☐ Geen addisionele gesondheidsprobleme om te verklaar

✐ ....

✐ ....

✐ ....

✐ ....

✐ ....

40. Dui asseblief u huidige "rookstatus" aan:

○ Nog nooit gerook nie
○ 'n voormalige roker
○ Huidige roker (sluit geleentheidsroker in)

Aktiwiteite en deelname

Die volgende gedeelte gaan oor probleme wat u in u lewe ondervind. Neem in aanmerking buide goeie sowel as die swak dae.

	In die laaste 4 weke, in hoe 'n mate het u 'n problem geondervind om…	*1 Geen probleem*	*2*	*3*	*4*	*5 Uiterste probleem*
**41.**	**… daaglikse roetine uit te voer?**	○	○	○	○	○
**42.**	**… stress te hanteer?**	○	○	○	○	○
**43.**	**… In hoe 'n mate besorg dinge wat met die hande en vingers gedoen moet word vir u probleme bv. om klein voorwerpe op te tel of om houers oop te maak?**	○	○	○	○	○
**44.**	**… te kom waar u wil wees?**	○	○	○	○	○
**45.**	**… publieke vervoer te gebruik?**	○	○	○	○	○
**46.**	**… privaate vervoer te gebruik?**	○	○	○	○	○
**47.**	**… na u gesondheid om te sien, gesond te eet, oefen of u medikasie te neem?**	○	○	○	○	○
**48.**	**… u huishoudelike take klaar te maak?**	○	○	○	○	○
**49.**	**… ander te versorg en van hulp te wees?**	○	○	○	○	○
**50.**	**… met ander te interakteer?**	○	○	○	○	○
**51.**	**… met intieme verhoudings?**	○	○	○	○	○
**52.**	**… dinge vir ontspanning of plesier te doen?**	○	○	○	○	○
**53.**	**… word u kort van asem gedurende fisieke inspanning?**	○	○	○	○	○
**54.**	**Is u in staat om te ongesteund te sit?**<br>○ Nee<br>○ Ja → Hoeveel van 'n probleem is dit om vir lang periodes soos 30 minute te sit**?**	○	○	○	○	○
**55.**	**Is u in staat om ongesteund te staan?**<br>○ Nee<br>○ Ja → Hoeveel van 'n probleem is dit om vir lang periodes soos 30 minute te staan**?**	○	○	○	○	○

Hierdie vrae gaan oor u vermoë om aktiwiteite wat basiese mobiliteit (beweeglikheid) vereis, te kan doen. Kies die respons wat u vermoë om sonder die hulp van ander, maar met hulp van die toerusting en apparate wat u normaalweg gebruik, die beste beskryf, bv. verplasinsplank, hyskraan, hospitaalbed.

	**Is u in staat om…**	*Geen moeilikheid*	*Kleine moeilikheid*	*Met sommige moelikheid*	*Met baie moeilikheid*	*Nie in staat om uitevoer nie*
**56.**	… vanaf ‘n posisie waar u op u rug lê, sonder hulp op te staan?	○	○	○	○	○
**57.**	…‘n swaar deur oop te stoot?	○	○	○	○	○
**58.**	… vanaf ‘n sittende posisie op die kant van die bed te verskuif deur op u rug te gaan lê?	○	○	○	○	○

Onafhanklikheid van aktiwiteite van die daaglikse lewe

Vir elke item moet u die stelling aftik wat u huidige toestand ten beste reflekteer. Lees asseblief die teks sorgvuldig deur en kies slegs een boks in elke seksie.

59. Eet en drink

- ○ Ek benodig kunsmatige voeding of 'n maagbuis.
- ○ Ek benodig algehele bystand met eet/ drink.
- ○ Ek benodig gedeeltelike bystand met eet/ drink of om met aanpassingsapparate aan- of uit te trek.
- ○ Ek eet/ drink onafhanklik, maar benodig aanpassingsapparate of hulp met die sny van voedsel, skink van drankies en oopmaak van houers.
- ○ Ek eet/ drink onafhanklik sonder hulp of aanpassingsapparate.

60. Was van bo-lyf en hoof

Die was van die bolyf en hoof sluit in seepsmeer en die afdroog en die gebruik van 'n waterkraan.

- ○ Ek benodig totale bystand.
- ○ Ek benodig gedeeltelike bystand.
- ○ Ek is onafhanklik maar benodig aanpassingsapparate of spesifieke toerusting (*bv. balke, stoel*)
- ○ Ek is onafhanklik en benodig geen aanpassingsapparate of spesifieke toerusting.

61. Was van onderlyf

Die was van die onderlyf sluit in seepsmeer en die afdroog en die gebruik van 'n waterkraan.

- ○ Ek benodig totale bystand.
- ○ Ek benodig gedeeltelike bystand.
- ○ Ek is onafhanklik maar benodig aanpassingsapparate of spesifieke toerusting (*bv. balke, stoel*)
- ○ Ek is onafhanklik en benodig geen aanpassingsapparate of spesifieke toerusting.

62. Klee (aantrek) van die bolyf

Die klee van die bolyf sluit in die aan- en uittrek van klere soos T-hemde, bloese hemde, brassieres, skawe en ortose(bv. armspalse, nekstutte, korsette)

- *Maklik- om – aan- te- trek klere is klere sonder knope, ritssluiters en veters*
- *Moeilik- om- aan- te- trek klere is klere met knope, ritssluiters en veters*

O Ek benodig algehele bystand.
O Ek benodig gedeeltelike bystand, selfs met maklik- om- aan- te- trek klere.
O Ek benodig nie hulp met maklik- om – aan- te- trek klere nie, maar gebruik aanpassingsapparate of spesifieke toerusting
O Ek is onafhanklik met maklik- om- aan- te- trek klere, maar benodig hulp of aanpassingsapparate of 'n spesifieke omgewing met moeilik- om- aan- te- trek klere.
O Ek is heeltemal onafhanklik.

63. Klee van die onder gedeelte van die liggaam

Die aantrek van die onder gedeelte van die liggaam sluit in die aan- en uittrek van klere soos kortbroeke, broeke, skoene sokkies, gordels en ortoses soos 'n beenspalk

- *Maklik- om – aan- te- trek klere is klere sonder knope, ritssluiters en veters*
- *Moeilik- om- aan- te- trek klere is klere met knope, ritssluiters en veters*

O Ek benodig algehele bystand.
O Ek benodig gedeeltelike bystand, selfs met maklik- om- aan- te- trek klere.
O Ek benodig nie hulp met maklik- om – aan- te- trek klere nie, maar gebruik aanpassingsapparate of spesifieke toerusting

O Ek is onafhanklik met maklik- om- aan- te- trek klere, maar benodig hulp of aanpassingsapparate of ‘n spesifieke omgewing met moeilik- om- aan- te- trek klere.
O Ek is heeltemal onafhanklik.

64. Selfversorging

Bv. aktiwiteite soos om hande te was, tande te borsel, hare te borsel, te skeer en grimering te doen.

- ○ Ek benodig volle hulp.
- ○ Ek benodig gedeeltelike hulp.
- ○ Ek is onafhanklik met aanpassingsapparate.
- ○ Ek is onafhanklik sonder aanpassingsapparate.

65. Blaashantering

Dink asseblief aan die manier hoe u u blaas verlig.

A. Gebruik van ‘n interne kateter

- ○ Ja → *Gaan asseblief na vraag nommer 66*
- ○ Nee → *Antwoord asseblief B and C.*

B. Onderbroke katetergebruik

- ○ Ek benodig algehele hulp
- ○ Ek doen dit self met bystand.
- ○ Ek doen dit self sonder bystand.
- ○ Ek gebruik dit nie

***C.* Die gebruik van eksterne dreineringsinstrumente** *bv. kondoomkateter, luiers, sanitêre doeke*

- ○ Ek benodig algehele bystand
- ○ Ek benodig gedeeltelike bystand
- ○ Ek gebruik dit sonder bystand
- ○ Ek is selfbeheerst met uriene en gebruik geen dreineringsinstrument nie.

66. Beheer van ontlasting

A. Het u hulp met ontlasting nodig *(bv om ‘n setpil te gebruik)***?**

- ○ Ja
- ○ Nee

B. Ek ontlas…

- ○ Onreëlmatig of weinig (minder as een keer in drie dae)
- ○ Gereeld(eenkeer of meer in drie dae)

C. Ontlastingsonbeheerstheid (ongelukkies) vind plaas .....

- ○ Daagliks
- ○ 1-6 times keer per week
- ○ 1-4 keer elke maand
- ○ Minder as een keer per maand
- ○ Nooit

67. Toiletgebruik

Dink asseblief aan die gebruik van die toilet, die was van jou genetalieë en hande, die aan- en uittrek van klere en die gebruik van sanitêre doekies en luiers..

- ○ Ek benodig algehele hulp

○ Ek benodig gedeeltelike hulp
○ Ek benodig gedeeltelike hulp, maar kan myself skoonmaak.
○ Ek het nie hulp nodig nie, maar wel aanpassingsapparate (bv. balke) of spesiale omgewing (bv.rolstoeltoeganklike toilet)
○ Ek benodig nie enige aanpassingsapparaat of spesiale omgewing nie.

68. Watter van die volgende aktiwitete kan u sonder hulp of elektroniese apparate doen?

Merk als wat van toepassing is

☐ Draai u bolyf in die bed.
☐ Draai u onderlyf in die bed.
☐ Sit op in die bed.
☐ Doen armopstote in `n stoel of rolstoel.
☐ Ek benodig hulp met al die aktiwiteite.

69. ○ **Verplasings van die bed na die rolstoel.**
○ Ek benodig algehele hulp
○ Ek benodig gedeeltelike hulp, toesig en aanpassingsapparate (bv. skuifplank)
○ Ek benodig nie hulp of aanpassingsapparate nie.
○ Ek gebruik nie 'n rolstoel nie.

70. Rondbeweeg oor gemiddelde distansies (10 tot 1000 meter)

Ek gebruik 'n rolstoel om rond te beweeg, …

○ Ek benodig volle hulp.
○ Ek benodig 'n elektriese rolstoel of gedeeltelike hulp om 'n gewone rystoel te opereer.
○ Ek opereer my gewone rystoel onafhanklik.

Ek stap gemiddelde distansies en ek...

○ Benodig toesig terwyl ek stap (met of sonder loopapparate).
○ Loop met 'n loopraam of krukke, swaai vorentoe met beide voete.
○ Loop met krukke of twee stoke deur een voet voor die ander te plaas.
○ Loop met een stok.
○ Loop met 'n beenortose (bv 'n beenspalk).
○ Loop sonder loophulpmiddels.

Werk

71. Wat was die benaming of title van u hoofberoep voor u spinale koordbesering?

○ Ek was werkloos voor my besering.
Die naam of title van my hoofberoep was soos volg: *(wees asseblief so spesifiek as moontlik bv nie net klerk nie, maar bankklerk, nie net bestuurder nie, maar verkoopsbestuurder).*

✐ ....

72. Het u beroepsrehabilitasie dienste ontvang na u spinale koordbesering?

bv. beroepsvoorligting, beroepsheropleiding,werkvaardigheidsopleiding

○ Ja
○ Nee

73. Na u ontslag van u aanvanklike binne- pasiënt rehabilitasie, hoe lank het dit geneem voor u u betaalde werk hervat het?

○ Ek het nooit na aanvanklike binne- pasiënt rehabilitasie weer gewerk nie.
○ Onmiddellik na aanvanklike rehabilitasie
○ Ek het my werk hervat na ....✐ .... jare en ✐ .... maande

74. Ontvang u tans 'n ongeskiktheidspensioen of 'n gelykstaande ongeskiktheidsvoordeel?

○ Ja
○ Nee

75. Wat is u huidige werksituasie?

Merk als wat van toepassing is

☐ Werk vir 'n loon of 'n salaries vir 'n werkgewer vir ✐ .... ure per week
☐ Werk vir 'n loon vir 'n werkgewer vir ✐ .... ure 'n week, maar tans met siekteverlof vir meer as 3 maande.
☐ Selfindiensname, werk vir ✐ .... ure 'n week.
☐ Werk as 'n onbetaalde familielid (werk in familiebesigheid)
☐ Huishoudster / Huishouer
☐ Student
☐ Werkklose
☐ Afgetree weens gesondheid
☐ Afgetree weens ouderdom
☐ Ander, spesifiseer asseblief: ✐ ....

76. Is u tans betrokke in betaalde werk?

○ Ja
○ Nee → *gaan asseblief na vraag 84*

77. Wat is die benaming of die title van u huidige hoofberoep?

Wees asseblief so spesifiek as moontlik bv. nie slegs klerk, maar bankklerk; nie slegs bestuurder, maar verkoopsbestuurder

✐ ....

78. Wil u meer, minder, of dieselfde hoeveelheid ure soos tans werk?

○ Meer ure
○ Minder ure
○ Dieselfde aantal ure

	1<br>Geen probleem	2	3	4	5<br>Uiterse probleem
**79. Hoeveel van ‘n probleem is dit om dit wat van u by die werk verwag word, gedaan te kry?**	○	○	○	○	○
**80. Hoeveel van ‘n probleem is dit om toegang tot u werkplek te verkry?** *bv. toegang tot die gebou, u kantoor of die toilette*	○	○	○	○	○

	Heeltemal	*Tot `n groot mate*	*Tot `n mate*	*Tot `n mindere mate*	*Glad nie*	*Ek het nie so `n behoefte nie*
**81. Beskik u oor die hulpverleningswerksapparate wat u nodig het om te werk?** *bv. hulpverleningsrekenaarapparate, arm- of handstutte of kunsmatige ledemate.*	○	○	○	○	○	○

Die volgende vrae hou verband met u huidige beroep of werk: Vir elkeen van die volgende stellings, dui asseblief aan of u daarmee ten volle saamstem, saamstem, nie saamstem nie of sterk daarmee verskil.

	Stem ten volle saam	*Stem saam*	*Stem nie saam nie*	*Verskil sterkliks*
**82. Ek ontvang die erkenning wat ek vir my werk verdien.**	○	○	○	○
**83. Al my harde werk en prestasies in ag geneem, is my vergoeding billik.** *→ gaan asseblief na vraag 87*	○	○	○	○

84. Sal u daarvan hou om `n betaalde werk te bekom?

- ○ Ja
- ○ Nee

85. Voel u in staat om betaalde werk te kan verrig?

- ○ Ja, vir 1 – 11 ure `n week
- ○ Ja, vir 12 – 20 ure `n week
- ○ Ja, vir meer as 20 ure `n week
- ○ Nee, glad nie

86. Om watter redes werk u tans nie?

Merk als wat van toepassing is

- ☐ Gesondheidstoestand of gestremdheid
- ☐ Besig met opvoedings- en werksopleiding
- ☐ Persoonlike familieverantwoordelikheid
- ☐ Vind nie geskikte werksgeleentheid nie
- ☐ Weet nie waar en hoe om werk te vind nie
- ☐ Het nie die finansiële behoefte nie
- ☐ Ouer of eggenoot weier dat ek werk
- ☐ Onvoldoende vervoerdienste
- ☐ Ontoeganklikheid tot moontlike werksplekke *bv. toegang tot die gebou, u kantoor of die toilette*
- ☐ Kom hulpverleningsapparate kort.
- ☐ Vrees dat u u ongeskiktheidsvoordeel sal verloor? *bv.pensioen, gesondheidsversekerigsdekking*
- ☐ Ek wil nie werk nie
- ☐ Ander, spesifiseer asb.: ✎ …………………………………………………………………………………..

Omgewingsfaktore

In die daagse lewe word ons aan talle eksterne invloede of omgewingsfaktore blootgestel. genaamd die sogenaamde omgewingsfaktore. Dit kan jou lewe vergemaklik of bemoeilik. Dink aan die laaste 4 weke en beoordeel asseblief hoe hierdie omgewingsfaktore u deelname in die gemeenskap/ samelewing beïnvloed het.

		Nie van toepassig	*Geen invloed*	*Maak my lewe tot n mate moeilik*	*Maak my lewe baie moeilik*
**87.**	**Afwesigheid of onvoldoende toeganklikheid tot openbare plekke**<br>*bv. ontoeganklike publieke geboue,parke*	○	○	○	○
**88.**	**Afwesigheid of onvoldoende toegang tot vriende en familie se huise.**	○	○	○	○
**89.**	**Swak klimaatstoestande**<br>*bv. weer,seisoen,temperatuur,humiditeit*	○	○	○	○
**90.**	**Negatiewe gesindhede van die gemeenskap teenoor gestremde persone**<br>*bv. vooroordeel, stigma, onkunde*	○	○	○	○
**91.**	**Negatiewe gesindhede van u gesin an ander familie teenoor u gestremdheid**<br>*bv. vooroordeel, gebrek aan ondersteuning, oorbeskerming*	○	○	○	○
**92.**	**Negatiewe gesindhede van u vriende teenoor u gestremdheid**<br>*bv. vooroordeel, gebrek aan ondersteuning, oorbeskerming*	○	○	○	○
**93.**	**Negatiewe gesindhede van u bure, kennisse en kollegas teenoor u gestremdheid**<br>*vooroordeel, gebrek aan ondersteuning, oorbeskerming*	○	○	○	○
**94.**	**Gebrek aan- of onvoldoende ondersteuningstegnologie om oor kort afstande te beweeg**<br>*gebrek aan- of onvoldoende hulp om trappe te klim, loop apparate, rolstoel*	○	○	○	○
**95.**	**Gebrek aan of onvoldoende aanpassing van vervoer oor lang afstande**<br>*bv. tekort aan aangepaste motor, publieke vervoer wat moeilik gebruik word.*	○	○	○	○
**96.**	**Tekort aan of onvoldoende verpleegsorg en ondersteunende dienste**<br>*bv. tekort aan of onvoldoende gesondheidssorg by die huis of persoonlike hulp*	○	○	○	○
**97.**	**Gebrek aan of onvoldoende medikasie en mediese bystand en -voorrade**<br>*bv. gebrek aan of onvoldoende kateters, ontsmettingsmiddels,spalke,kussings*	○	○	○	○
**98.**	**Moeilike finansiële posisie**<br>*bv. tekort aan geld*	○	○	○	○

99.	**Gebrek aan- of tekort aan kommunikasieapparate** *bv. gebrek aan of onvoldoende skryfapparate,rekenaar,telefoon, muis*	○	○	○	○
100.	**Gebrek aan of onvoldoende staatsdienste** *bv. gebrek aan of onvoldoende ongeskiktheidsversekering of ander voordele*	○	○	○	○

Gesondheidsorg dienste

101. Wie was die gesondheidsorg verskaffers wat u besoek het, of wie u huis besoek het, in die laaste 12 maande?

Merk als wat van toepassing is

- ☐ Primêre sorg dokter/ algemene praktisyn
- ☐ Rehabilitasie dokter / spinale koord beseringsdokter
- ☐ Ander spesialisasie dokter *e.g., Chirurg, ginekoloog, psigiater, oogaarts*
- ☐ Verpleegster of vroedvrou
- ☐ Tandarts
- ☐ Fisioterapeut
- ☐ Chiropraktisyn
- ☐ Arbeidsterapeut
- ☐ Sielkundige
- ☐ Tradisionele geneeskundige *e.g., naturopaat, acupuncturist,kruiedokter*
- ☐ Apteker
- ☐ Huis gesondheidsorg werker
- ☐ Ander, spesifiseer asseblief……. ✎ ……………………………………………………………………
- ☐ Ek het geen gesondheidssorgvoorsiener gedurende die laaste 12 maande besoek nie.

102. Oor die afgelope 12 maande hoeveel keer was u 'n pasiënt in 'n hospital, rehabilitasiefasiliteit of 'n ander versorgingsfasiliteit vir ten minste een nag?

✎ …………… *(aantal kere)*

	**Hoe sal u die volgende beoordeel na aanleiding van u laaste besoek aan 'n gesondheidssorgvoorsiener?**	*Baie goed*	*Goed*	*Nie goed of swak nie*	*Swak*	*Baie swak*
103.	**...u ervaring om met respek behandel te word.**	○	○	○	○	○
104.	**…hoe duidelik die gesondheidssorgvoorsieners dinge verduidelik.**	○	○	○	○	○
105.	**… u ervaring van u betrokkenheid in die besluite wat geneem word rakende u behandeling.**	○	○	○	○	○

106. In die afgelope 12 maande, het u gesondheidssorg benodig, maar dit nie ontvang nie?

- ○ Nee
- ○ Ja. Watter redes verduidelik ten beste waarom u nie die gesondheidssorg ontvang het wat u nodig gehad het nie?.

Merk als wat van toepassing is

- ☐ Ek kon nie die besoek bekostig nie.
- ☐ Daar was geen diens nie.
- ☐ Geen vervoer beskikbaar nie.
- ☐ Ek kon nie die vervoerkoste bekostig nie.
- ☐ Ek was voorheen swak behandel.

- ☐ Ek kon nie die tyd afneem nie of het ander verpligtinge gehad.
- ☐ Die gesondheidssorgvoorsiener het 'n tekort aan geneesmiddels en toerusting gehad.
- ☐ Die gesondheidssorgvoorsiener se vaardighede was ontoereikend.
- ☐ Ek het nie geweet waarheen om te gaan nie.
- ☐ Ek het probeer, maar is gesondheidssorg geweier.
- ☐ Ek het gedink dat ek nie siek genoeg was nie.
- ☐ Ander, spesifiseer asseblief ✎ ....

	Very satisfied	*Satisfied*	*Neither satisfied nor dissatisfied*	*Dissatisfied*	*Very dissatisfied*
**107. In die algemeen, hoe tevrede is u met die gesondheidssorgdienste in u area?**	○	○	○	○	○

Persoonlike faktore

Die volgende vrae handel oor hoe u uself sien.

	1 Glad nie	*2*	*3*	*4*	*5 Ten volle*
**108. In hoe 'n mate is u seker dat u oor die vermoë beskik om u sin te kry as iemand u sou teenstaan?**	○	○	○	○	○
**109. Hoe seker is u dat u in staat is om onverwagte gebeurlikhede effektief te kan hanteer?**	○	○	○	○	○
**110. Hoe seker is u dat u kontak sal kan behou met die mense wat vir u belangrik is?**	○	○	○	○	○
**111. Hoe seker is u dat u goeie gesondheid kan handhaaf?**	○	○	○	○	○
**112. Dink jy dat jou spinale koordbesering u 'n sterker persoon gemaak het?**	○	○	○	○	○
**113. Bekommer u u oor wat met u in die toekoms kan gebeur?** *Bv. dink aan om na u self om te sien of om 'n oorlas vir ander te wees.*	○	○	○	○	○
**114. Voel u dat u in staat is om u drome, verwagtinge en wense te verwesenlik?**	○	○	○	○	○
**115. Is u in staat om belangrike besluite in die lewe te kan maak?** *bv. om te besluit waar en by wie om te woon en hoe om u geld te spandeer.*	○	○	○	○	○
**116. Voel u dat ander u insluit as u met hulle is?**	○	○	○	○	○

117. **Het u in die laaste 12 maande enige groot negatiewe lewensgebeurlikheid beleef,**
bv. 'n ernstige konfliksituasie met ander of egskeiding of die dood van 'n geliefde.

- ○ Nee
- ○ Ja, spesifiseer asseblief: ✎ ....

Lewenskwaliteit en algemene gesondheid

Die volgende vrae behels u beoordeling van u kwaliteit van lewe oor die laaste 14 dae. Dink asseblief aan u lewe gedurende die laaste 14 dae. Hou asseblief die volgende in gedagte: u standaarde, verwagtinge, genietinge en verse.

In die laaste 14 dae	*Baie swak*	*Swak*	*Nie swak of goed nie*	*Goed*	*Baie goed*
118. **Hoe sal u u kwaliteit van lewe beoordeel?**	○	○	○	○	○

	Baie ontevrede	*Ontevrede*	*Nie tevrede of ontevrede nie*	*Tevrede*	*Baie tevrede*
119. **In hoe 'n mate is u tevrede met u gesondheid?**	○	○	○	○	○
120. **In hoe 'n mate is u tevrede met u vermoë om u daaglikse aktiwiteite uit te voer?**	○	○	○	○	○
121. **In hoe 'n mate is u tevrede met uself?**	○	○	○	○	○
122. **In hoe 'n mate is u tevrede met u persoonlike verhoudings?**	○	○	○	○	○
123. **In watter mate is u tevrede met u lewensomstandighede?**	○	○	○	○	○

124. **Hoe sal u u algemene gesondheid beskryf?**

- ○ Puik
- ○ Baie goed
- ○ Goed
- ○ Gemiddeld
- ○ Swak

125. **In vergeleiking met 'n jaar te vore, hoe sal u tans u algemene gesondheid beskryf?**

- ○ Baie beter
- ○ `n bietjie beter
- ○ Feitlik dieselfde
- ○ Ietwat verswak
- ○ Baie verswak

Ons bedank u grootliks vir u deelname in die InSCI opname!
