# Supplementary material for: The Role of Environmental Factors on Health Conditions, General Health and Quality of Life in Persons with Spinal Cord Injuries in South Africa
Source: Int J Environ Res Public Health. 2023 May 3;20(9):5709. doi: 10.3390/ijerph20095709 (PMC10178437; doi:10.3390/ijerph20095709)
Supplement: Supplementary file 1 [file ijerph-20-05709-s001.zip › ijerph-2310147-supplementary.pdf]

## Supplementary material:

**Table S1:** Linear Regression model showing the association between Environmental factors with Health conditions (Secondary health conditions, Pain Intensity and Mental Health) adjusted for age, sex and severity of SCI.

| | **Secondary conditions** | | **Pain Intensity** | | **Mental Health** | |
|---|---|---|---|---|---|---|
| | B (95% CI) | p-value | B (95% CI) | p-value | B (95% CI) | p-value |
| **Environmental factors Sum score** | 0.55 (0.61 – 0.95) | <.001 | 0.16 (0.01 – 0.13) | 0.03 | -0.21 (-0.13 - -0.02) | .005 |
| **Environmental factors individual items** | | | | | | |
| **Public access** | | | | | | |
| No Influence/Not applicable | Reference | | Reference | | Reference | |
| Made my life a little harder | 0.08 (-1.31 – 4.11) | .310 | -0.02 (-0.76 – 0.98) | .808 | -0.10 (-1.16 – 0.33) | .273 |
| Made my life a lot harder | 0.30 (2.94 – 9.57) | <.001 | 0.05 (-0.74 – 1.40) | .547 | -0.08 (-1.33 – 0.48) | .358 |
| **Access at home** | | | | | | |
| No Influence/Not applicable | Reference | | Reference | | Reference | |
| Made my life a little harder | 0.08 (-1.31 – 4.13) | .308 | 0.07 (-0.50 – 1.19) | .423 | -0.06 (-0.97 – 0.47) | .498 |
| Made my life a lot harder | 0.11 (-1.17 – 5.50) | .202 | 0.06 (-0.69 – 1.38) | .510 | -0.01 (-0.95 – 0.82) | .888 |
| **Climate** | | | | | | |
| No Influence/Not applicable | Reference | | Reference | | Reference | |
| Made my life a little harder | 0.04 (-1.94 – 3.17) | .636 | -0.10 (-1.37 – 0.25) | .178 | -0.35 (-0.79 – 0.56) | .735 |
| Made my life a lot harder | 0.06 (-2.57 – 5.34) | .491 | -0.04 (-1.49 – 0.94) | .655 | 0.02 (-0.92 – 1.18) | .807 |
| **Short transport** | | | | | | |
| No Influence/Not applicable | Reference | | Reference | | Reference | |
| Made my life a little harder | 0.08 (-1.18 – 3.85) | .297 | 0.03 (-0.64 – 1.00) | .667 | -0.07 (-1.03 – 0.37) | .353 |
| Made my life a lot harder | 0.33 (4.24 – 11.00) | <.001 | 0.08 (-0.54 – 1.66) | .317 | -0.12 (-1.67 – 0.20) | .124 |
| **Long transport** | | | | | | |
| No Influence/Not applicable | Reference | | Reference | | Reference | |
| Made my life a little harder | 0.14 (-0.66 – 4.75) | .138 | -0.02 (-0.99 – 0.73) | .771 | 0.01 (-0.71 – 0.77) | .934 |
| Made my life a lot harder | 0.25 (1.96 – 7.89) | .001 | -0.08 (-1.41 – 0.47) | .326 | 0.04 (-0.59 – 1.03) | .593 |
| **Appliance** | | | | | | |
| No Influence/Not applicable | Reference | | Reference | | Reference | |
| Made my life a little harder | 0.05 (-1.73 – 3.34) | .533 | -0.01 (-0.85 – 0.72) | .869 | -0.14 (-1.28 – 0.06) | .072 |
| Made my life a lot harder | 0.05 (-2.57 – 5.50) | .475 | -0.04 (-1.53 – 0.97) | .655 | 0.01 (-1.12 – 1.00) | .905 |
| **Finances** | | | | | | |
| No Influence/Not applicable | Reference | | Reference | | Reference | |
| Made my life a little harder | 0.01 (-3.08 – 3.48) | .904 | -0.13 (-1.69 – 0.33) | .187 | -0.08 (-1.21 – 0.51) | .422 |
| Made my life a lot harder | 0.08 (-1.76 – 4.52) | .388 | -0.12 (-1.58 – 0.36) | .217 | -0.22 (-1.76 – -0.11) | .026 |
| **Politics** | | | | | | |

| | | | | | | |
|---|---|---|---|---|---|---|
| No Influence/Not applicable | Reference | | Reference | | Reference | |
| Made my life a little harder | 0.04 (-1.94 – 3.43) | .586 | -0.10 (-1.32 – 0.34) | .246 | -0.09 (-1.10 – -0.32) | .281 |
| Made my life a lot harder | 0.03 (-3.03 – 4.43) | .712 | -0.02 (-1.32 – 0.99) | .779 | -0.03 (-1.15 – 0.83) | .749 |
| **Social attitudes** | | | | | | |
| No Influence/Not applicable | Reference | | Reference | | Reference | |
| Made my life a little harder | 0.03 (-2.16 – 3.09) | .727 | -0.03 (-0.99 – 0.65) | .681 | -0.11 (-1.23 – 0.17) | .135 |
| Made my life a lot harder | 0.13 (-0.55 – 7.80) | .088 | 0.50 (-0.87 – 1.73) | .514 | -0.04 (-1.41 – 0.81) | .596 |
| **Family attitudes** | | | | | | |
| No Influence/Not applicable | Reference | | Reference | | Reference | |
| Made my life a little harder | 0.02 (-2.27 – 2.93) | .805 | 0.00 (-0.83 – 0.79) | .955 | -0.03 (-0.81 – 0.56) | .714 |
| Made my life a lot harder | 0.12 (-0.98 – 8.80) | .116 | 0.04 (-1.11 – 1.93) | .598 | -0.16 (-2.66 – -0.09) | .037 |
| **Friends attitudes** | | | | | | |
| No Influence/Not applicable | Reference | | Reference | | Reference | |
| Made my life a little harder | 0.00 (-2.57 – 2.65) | .974 | 0.02 (-0.75 – 0.91) | .844 | -0.11 (-1.20 – 0.20) | .161 |
| Made my life a lot harder | 0.22 (2.49 – 12.07) | .003 | 0.03 (-1.26 – 1.78) | .738 | -0.14 (-2.49 – 0-08) | .065 |
| **Colleagues attitudes** | | | | | | |
| No Influence/Not applicable | Reference | | Reference | | Reference | |
| Made my life a little harder | 0.16 (0.46 – 6.01) | .023 | 0.03 (-0.71 – 1.08) | .682 | -0.12 (-1.38 – 0.14) | .112 |
| Made my life a lot harder | 0.27 (4.47 – 14.18) | <.001 | 0.08 (-0.76 – 2.38) | .308 | -0.10 (-2.28 – 0.39) | .162 |
| **Service** | | | | | | |
| No Influence/Not applicable | Reference | | Reference | | Reference | |
| Made my life a little harder | 0.15 (0.00 – 5.21) | .050 | 0.02 (-0.73 – 0.90) | .840 | -0.05 (-0.94 – 0.46) | .505 |
| Made my life a lot harder | 0.13 (-0.50 – 7.49) | .082 | 0.09 (-0.49 – 2.00) | .233 | 0.00 (-1.10 – 1.03) | .943 |
| **Communication** | | | | | | |
| No Influence/Not applicable | Reference | | Reference | | Reference | |
| Made my life a little harder | -0.03 (-3.34 – 2.14) | .664 | -0.12 (-1.54 – 0.17) | .113 | -0.16 (-1.49 – -0.04) | .039 |
| Made my life a lot harder | 0.15 (0.14 – 7.28) | .042 | -0.02 (-1.24 – 1.00) | .827 | -0.02 (-1.07 – 0.82) | .791 |

**Table S2:** Logistic Regression model showing the association between Environmental factors with General Health and QoL adjusted for age, sex and severity of SCI.

| | General Health | | QOL | |
|---|---|---|---|---|
| | OR (95% CI) | p-value | OR (95% CI) | p-value |
| **Environmental factors sum score** | 0.93 (0.88 – 0.99) | .016 | 0.93 (0.87 – 1.01) | .066 |
| **Environmental factors individual items** | | | | |
| **Public access** | | | | |
| No Influence/Not applicable | Reference | | Reference | |
| Made my life a little harder | 1.06 (0.52 – 2.16) | .866 | 1.17 (0.38 – 3.63) | .790 |
| Made my life a lot harder | 2.67 (0.98 – 7.28) | .055 | 1.39 (0.39 – 5.00) | .613 |
| **Access at home** | | | | |
| No Influence/Not applicable | Reference | | Reference | |
| Made my life a little harder | 1.28 (0.63 – 2.59) | .502 | 1.42 (0.46 – 4.34) | .542 |
| Made my life a lot harder | 0.99 (0.42 – 2.34) | .973 | 1.52 (0.42 – 5.57) | .525 |
| **Climate** | | | | |
| No Influence/Not applicable | Reference | | Reference | |
| Made my life a little harder | 0.98 (0.52 – 1.88) | .962 | 1.48 (0.55 – 4.00) | .439 |
| Made my life a lot harder | 1.68 (0.53 – 5.31) | .374 | 0.83 (0.16 –4.46) | .832 |
| **Short transport** | | | | |
| No Influence/Not applicable | Reference | | Reference | |
| Made my life a little harder | 1.19 (0.60 – 2.33) | .619 | 1.83 (0.66 – 5.09) | .246 |
| Made my life a lot harder | 1. 23 (0.48 – 3.21) | .666 | 0.70 (0.14 – 3.62) | .673 |
| **Long transport** | | | | |
| No Influence/Not applicable | Reference | | Reference | |
| Made my life a little harder | 1.55 (0.76 – 3.16) | .230 | 1.45 (0.47 – 4.45) | .522 |
| Made my life a lot harder | 2.20 (0.96 – 5.06) | .063 | 1.74 (0.54 – 5.61) | .353 |
| **Appliance** | | | | |
| No Influence/Not applicable | Reference | | Reference | |
| Made my life a little harder | 0.78 (0.41 – 1.48) | .443 | 2.49 (0.82 – 7.55) | .106 |
| Made my life a lot harder | 1.97 (0.58 – 6.69) | .275 | 2.34 (0.50 – 10.89) | .279 |
| **Finances** | | | | |
| No Influence/Not applicable | Reference | | Reference | |
| Made my life a little harder | 1.61 (0.69 – 3.73) | .270 | 2.08 (0.52 – 8.25) | .298 |
| Made my life a lot harder | 1.27 (0.57 – 2.81) | .557 | 1.51 (0.37 – 6.20) | .566 |
| **Politics** | | | | |
| No Influence/Not applicable | Reference | | Reference | |
| Made my life a little harder | 1.03 (0.52 – 2.06) | .932 | 1.25 (0.45 – 3.46) | .664 |
| Made my life a lot harder | 1.37 (0.51 – 3.67) | .530 | 0.30 (0.03 – 2.53) | .266 |
| **Social attitudes** | | | | |
| No Influence/Not applicable | Reference | | Reference | |
| Made my life a little harder | 0.97 (0.49 – 1.92) | .937 | 1.17 (0.40 – 3.43) | .781 |
| Made my life a lot harder | 0.85 (0.28 – 2.61) | .771 | 2.79 (0.75 – 10.40) | .127 |
| **Family attitudes** | | | | |
| No Influence/Not applicable | Reference | | Reference | |
| Made my life a little harder | 1.39 (0.70 – 2.73) | .345 | 0.75 (0.25 – 2.24) | .609 |
| Made my life a lot harder | 1.05 (0.29 – 3.78) | .940 | 1.63 (0.32 – 8.39) | .562 |
| **Friends attitudes** | | | | |
| No Influence/Not applicable | Reference | | Reference | |
| Made my life a little harder | 1.33 (0.67 – 2.65) | .416 | 0.78 (0.26 – 2.30) | .650 |
| Made my life a lot harder | 0.99 (0.27 – 3.58) | .988 | 0.71 (0.08 – 5.96) | .749 |
| **Colleagues attitudes** | | | | |
| No Influence/Not applicable | Reference | | Reference | |
| Made my life a little harder | 1.03 (0.48 – 2.20) | .937 | 1.76 (0.61 – 5.04) | .294 |
| Made my life a lot harder | 1.72 (0.41 –7.29) | .460 | 0.87 (0.10 – 7.50) | .901 |
| **Service** | | | | |
| No Influence/Not applicable | Reference | | Reference | |
| Made my life a little harder | 1.23 (0.63 – 2.40) | .541 | 0.99 (0.33 – 2.95) | .985 |
| Made my life a lot harder | 6.06 (1.29 – 28.38) | .022 | 2.32 (0.63 – 8.47) | .204 |
| **Communication** | | | | |
| No Influence/Not applicable | Reference | | Reference | |
| Made my life a little harder | 0.97 (0.48 – 1.96) | .940 | 0.59 (0.18 – 1.90) | .373 |

| | | | | |
|---|---|---|---|---|
| Made my life a lot harder | 3.30 (1.05 – 10.41) | .042 | 0.23 (0.03 – 1.82) | .163 |
